# Anemia, Micronutrient Deficiencies, and Malaria in Children and Women in Sierra Leone Prior to the Ebola Outbreak - Findings of a Cross-Sectional Study

**DOI:** 10.1371/journal.pone.0155031

**Published:** 2016-05-10

**Authors:** James P Wirth, Fabian Rohner, Bradley A Woodruff, Faraja Chiwile, Hannah Yankson, Aminata S Koroma, Feimata Russel, Fatmata Sesay, Elisa Dominguez, Nicolai Petry, Setareh Shahab-Ferdows, Mercedes de Onis, Mary H Hodges

**Affiliations:** 1 GroundWork, Crans-près-Céligny, Switzerland; 2 UNICEF, Freetown, Sierra Leone; 3 WHO, Freetown, Sierra Leone; 4 Ministry of Health and Sanitation, Freetown, Sierra Leone; 5 Helen Keller International, Freetown, Sierra Leone; 6 WHO West Africa, Ouagadougou, Burkina Faso; 7 USDA/ARS Western Human Nutrition Research Center, Davis, United States of America; 8 WHO Headquarters, Geneva, Switzerland; London School of Hygiene and Tropical Medicine, UNITED KINGDOM

## Abstract

To identify the factors associated with anemia and to document the severity of micronutrient deficiencies, malaria and inflammation, a nationally representative cross-sectional survey was conducted. A three-stage sampling procedure was used to randomly select children <5 years of age and adult women from households in two strata (urban and rural). Household and individual data were collected, and blood samples from children and women were used to measure the prevalence of malaria, inflammation, and deficiencies of iron, vitamin A, folate, and vitamin B_12_. 839 children and 945 non-pregnant women were included in the survey. In children, the prevalence rates of anemia (76.3%; 95% CI: 71.8, 80.4), malaria (52.6%; 95% CI: 46.0, 59.0), and acute and chronic inflammation (72.6%; 95% CI: 67.5, 77.1) were high. However, the prevalence of vitamin A deficiency (17.4%; 95% CI: 13.9, 21.6) was moderate, and the prevalence of iron deficiency (5.2%; 95% CI: 3.3, 8.1) and iron-deficiency anemia (3.8%; 95% CI: 2.5, 5.8) were low. Malaria and inflammation were associated with anemia, yet they explained only 25% of the population-attributable risk. In women, 44.8% (95% CI: 40.1, 49.5), 35.1% (95% CI: 30.1, 40.4), and 23.6% (95% CI: 20.4, 27.3) were affected by anemia, malaria, or inflammation, respectively. The prevalence rates of iron deficiency (8.3%; 95% CI: 6.2, 11.1), iron-deficiency anemia (6.1%; 95% CI: 4.4, 8.6), vitamin A deficiency (2.1%; 95% CI: 1.1, 3.1) and vitamin B_12_ deficiency (0.5%; 95% CI: 0.2, 1.4) were low, while folate deficiency was high (79.2%; 95% CI: 74.1, 83.5). Iron deficiency, malaria, and inflammation were significantly associated with anemia, but explained only 25% of cases of anemia. Anemia in children and women is a severe public health problem in Sierra Leone. Since malaria and inflammation only contributed to 25% of anemia, other causes of anemia, such as hemoglobinopathies, should also be explored.

## Introduction

Anemia, defined as low hemoglobin concentration, is estimated to globally affect 43% of children under five years of age, 29% of non-pregnant women (aged 15–49 years), and 38% of pregnant women. These estimates are considerably higher for Central and West Africa (71%, 48%, and 56%, respectively) [1]. The consequences of anemia vary depending on the severity and the population group, but include reduced work capacity, poor pregnancy outcomes, increased maternal and perinatal mortality and morbidity, impaired cognitive performance and poorer educational achievement [2]. Its causes are multifactorial, particularly in sub-Saharan Africa, and may include iron deficiency, malaria, helminthiasis, schistosomiasis, hemoglobinopathies, and deficiencies of other micronutrients [3,4]. In areas with high exposure to acute and chronic infection, inflammation may also be a common cause, albeit often difficult to fully separate from other factors [5].

Sierra Leone is one of the poorest countries in the world with slow economic growth despite increasing political stability [6]. Sierra Leone has also recently gained additional international attention due to the devastating Ebola outbreak, starting in May 2014 [7]. Anemia prevalence is very high among Sierra Leonean children <5 years of age and women of reproductive age [1], and nearly one-third of children are stunted affects (28.8%,SLNNS 2014) [8], indicating poor nutritional and health status. Data on specific micronutrient deficiencies are very scarce and are needed to design and implement nutrition and public health programs aimed at reducing micronutrient deficiencies and anemia. The Sierra Leone Micronutrient Survey (SLMS), reported in this paper, was implemented to assess the magnitude of micronutrient deficiencies in Sierra Leone, identify the factors associated with anemia, and establish a “baseline” which can be used to gauge the progress of large-scale health and nutrition programs targeted at children and women.

## Methods

### Ethics and consent

The survey protocol, questionnaires (S1 Appendix), and consent forms were approved by the Office of the Sierra Leone Ethics and Scientific Review Committee, Directorate of Training, Non-Communicable Diseases and Research, Connaught Hospital, Ministry of Health and Sanitation. Oral consent was sought for the respondent of the household interview, which only contained questions related to household demographics (e.g. household roster) and socioeconomic status. Written informed consent (i.e. signature) was received for blood sampling from selected pregnant and non-pregnant women and the caregivers of selected children. If the respondent could not read or write, the consent form was read aloud to him/her and a thumb print using indelible ink was taken in place of a signature. Survey respondents diagnosed with current or recent malaria or severe anemia were referred for further diagnosis and treatment at the local health facility. No blood was taken from children younger than 6 months of age to avoid injury and undue stress to mothers.

### Study design and participants

Data collection for the Sierra Leone Micronutrient Survey (SLMS) was conducted in November and December 2013. The SLMS was a cross-sectional national survey designed to produce estimates of micronutrient deficiencies in children aged 6–59 months and women of reproductive age for two strata—urban and rural. Two- or three-stage sampling was carried out, depending on the target group. Using a random starting point, 30 enumeration areas (EA) were selected probability proportional to population size within each stratum (60 EAs in total). Twenty-four households were then randomly selected in each EA from a list of all households in an EA compiled just prior to the field work. Within a household, all pregnant women were enrolled, followed by an additional stage of sampling to select one child <5 years old and one non-pregnant women using a Kish table [9]. If the mother of a selected child was not randomly selected, she was purposefully enrolled in the survey to allow later investigation of associations between maternal and child factors.

### Data collection procedures

Prior to data collection, all field workers (supervisors, team leaders, interviewers, phlebotomists, and lab technicians) participated in a one-week training on proper data and specimen collection procedures. The training consisted of two days of theoretical training and one day of role play to familiarize field workers with the survey procedures, instruments, and equipment. As part of the role play, phlebotomists drew blood specimens from field workers, and laboratory technicians practiced processing and labeling samples. At the end of the training, a written test was administered to team members. As more trainees were recruited than were needed, only the best performing individuals were selected for actual data collection, and the best performing and most experienced interviewers were hired as team leaders. Following the classroom training, two-days of field testing in two EAs not included in the SLMS (one urban and one rural EA) was conducted to familiarize teams with logistical procedures, team dynamics, and real-world scenarios.

As part of the SLMS data collection, a household questionnaire was administered first, followed by the child and women questionnaires. If present, the head of the household responded to the household questionnaire, and if not present, another adult in the household responded. The selected woman responded to the women questionnaire, and the caretaker of the selected child provided responses to the child questionnaire. Questionnaires were administered in either Krio, Themne, Mende, or English, depending on the mother tongue of the respondent. If the required individuals were not present at the household during the first visit, at least two follow up visits were made to interview the selected household or individual.

The household questionnaire contained modules related to household demographics, socio-demographic variables, water, sanitation and hygiene, and household consumption of wheat flour and vegetable oil. In addition, each household was requested to provide a salt sample for the quantitative testing of iodine concentrations using colorimetric method on the iCheck Iodine^™^ analyzer (Bioanalyt GmbH, Teltow, Germany) [10]. In addition, pregnant and non-pregnant women were requested to provide a urine sample for the assessment of iodine status using the ammonium persulfate/Sandell-Kolthoff reaction method [11]. The salt and urinary iodine results have been analyzed separately and are presented elsewhere [12].

Children and women were invited for blood sampling in a nearby phlebotomy site established for the survey. Non-fasting blood was drawn in an EDTA-coated tube from children and non-pregnant women; for pregnant women, only a few drops of blood were collected to measure hemoglobin concentration and current and/or recent malaria status. Hemoglobin concentration was measured using a portable hemoglobinometer (Hb201+, HemoCue AB, Ängelholm, Sweden) and malaria status was checked using a dipstick antigen rapid diagnostic test (Paracheck Pf^™^, Orchid Biomedical systems, Goa, India). The malaria test assess if the blood contains histidine-rich protein-2, a protein found in the blood stream during a current malaria episode and remains in the blood stream a up to one month after infection has passed [13]. Thus the test measures the presence of both a current or a recent malaria infection.

The remaining blood from children and non-pregnant women was stored and transported cold (between 1–8°C) and protected from light. Later in the day, these samples were centrifuged and the plasma was separated and frozen at -20°C. This temperature was maintained during domestic transport and sample sorting. Samples were packed on dry ice (-70°C) during shipment to international laboratories where they were analyzed for iron, vitamin A, folate, vitamin B_12_, and inflammation markers.

Specifically, plasma was analyzed in one run for retinol-binding protein (RBP), ferritin, C-reactive protein (CRP) and a1-acid glycoprotein (AGP) at the VitMin-Lab (Wilstaett, Germany) using the sandwich ELISA method of Erhardt et al [14]. In addition, plasma from non-pregnant women was sent to the USDA/ARS Western Human Nutrition Research Center (Davis, USA) for analysis of plasma folate and vitamin B_12_ concentrations using the Cobas e411 analyzer (Roche Diagnostics USA). Because of concerns that Ebola virus may be present in some samples, samples analyzed for plasma folate and vitamin B_12_ were heated to 60°C for 60 minutes. This same heating procedure was conducted on 32 samples with known values and only a negligible deterioration in folate or vitamin B_12_ was observed. The coefficient of variation between non-heated and heated folate and vitamin B_12_ samples was 5.5% and 3.2%, respectively.

### Deficiency cut-offs

In children and pregnant women, hemoglobin concentrations <70 g/L defined severe anemia, 70–99 g/L defined moderate anemia, and 100–109 g/L defined mild anemia. In non-pregnant women, anemia was defined as hemoglobin<120 g/L; hemoglobin <80 g/L, 80–109 g/L, 110–119 g/L denoted severe, moderate, and mild anemia, respectively [15]. To classify population anemia status, World Health Organization (WHO) definitions of the public health severity of anemia were also used [15]. For both children and non-pregnant women, ferritin concentrations were adjusted for elevated AGP and CRP according to the procedure recommended by Thurnham [16]. In children, iron deficiency was defined as plasma ferritin <12 μg/L; while in non-pregnant women iron deficiency was <15 μg/L [17]. Iron deficiency anemia consisted of coexistent anemia and iron deficiency. RBP concentrations were adjusted for elevated AGP and CRP [18], and vitamin A deficiency in children and women was defined as RBP <0.7μmol/L and vitamin A insufficiency in women defined as RBP <1.05μmol/L [19]. Folate deficiency was defined as plasma folate <10nmol/L, and vitamin B_12_ deficiency was defined as a plasma concentration <150pmol/L [20]. Inflammation status was classified into four categories: no inflammation with normal CRP and AGP, elevated CRP (>5mg/L) alone defined incubation, elevated CRP and AGP (>1.0 g/L) defined early convalescence, and elevated AGP alone defined late convalescence. In addition, elevated CRP and/or AGP were used to identify individuals as having *any* inflammation.

### Data management and statistical analysis

All field data were doubly entered using a pre-programmed data-entry screen (CSPro v. 5.0) and then merged and cross-checked. Laboratory data were either auto-generated or doubly entered (Microsoft Excel, version 2010). Data analysis was done using SPSS version 22 with the complex survey module. Statistical weighting accounted for the unequal selection probability in the two strata. Standardized sampling weights were calculated for each stratum by dividing the overall sampling fraction for the entire survey sample by the sampling fraction within that stratum. Histograms and the Kolmogorov-Smirnov test were used to check for normality of data distribution. The statistical precision of all prevalence and mean estimates were was assessed using 95% confidence limits, which were calculated accounting for the complex sampling. The independent samples median and Kruskal-Wallis tests were used for to compare of groups of non-normal data. For binomial data, the chi^2^ test was applied.

Child age was initially calculated in days by subtracting the child's date of birth from the date of interview. Subsequently the child's age in months was calculated using an average number of 30.4375 days per month. Pregnant women were identified during the household interview, and pregnancy status was confirmed by self report during the interview with each selected woman.

Using data on household characteristics and assets, principal component analysis was used to calculate an index of household wealth; households were subsequently classified into wealth quintiles base on approaches employed by UNICEF MICS, the World Bank, and the World Food Programme [21,22]. WHO classifications were used to define household water source as “safe” or “unsafe” and sanitation facilities as “improved” or “unimproved” [23]. The population attributable risk for anemia was calculated using the method proposed by Diaz-Quijano to derive relative risk estimates from logistic regression models [24]. These relative risks were then used to calculate population attributable risks for the independent variables significantly associated with anemia in the child and women regression models.

## Results

### Households

The household response rate was 97%, leading to completed household interviews of 1,354 households. Table 1 shows the distributions of household characteristics and, for children and women, demographic characteristics and prevalence of anemia, micronutrient deficiencies, inflammation, and malaria status. Six out of ten households surveyed were located in rural areas, and one-third of households were located in the North region. Households were relatively large and nearly three-quarters were headed by men. Approximately 70% of household heads had either no formal education or had attended only primary school or less. Nearly all households used natural cooking fuels, such as wood, charcoal, and coal. Almost two-thirds of households had “unimproved” sanitation facilities, and almost one-quarter regularly drank water from an “unsafe” source.

**Table 1:** Characteristics of surveyed households and micronutrient status of children 6–59 months of age and women of reproductive age, by national and urban/rural residence, Sierra Leone 2013.

Characteristic	% / mean ^a^	95%CI ^b^
**Households (n = 1,363)**		
**Residence**		
Rural, %	60.4	(59.5, 61.3)
Urban, %	39.6	(38.7, 40.5)
**Region**		
East, %	22.7	(13.4, 35.9)
North, %	33.4	(22.1, 46.9)
South, %	25.7	(15.8, 39.0)
West, %	18.2	(10.8, 28.9)
**Household size**		
Mean	6.8	(6.45, 7.21)
Median	5.5	-
**Sex of head of household**		
Male, %	72.7	(69.2, 75.9)
Female, %	27.3	(24.1, 30.8)
**Education of head of household number**		
None, %	59.2	(54.5, 63.7)
Primary school or less, %	11.1	(9.3, 13.2)
Secondary or above, %	29.7	(25.5, 34.3)
**Use of cooking fuel** ^c^		
Clean, %	0.2	(0.1, 0.5)
Natural, %	99.8	(99.5, 99.9)
**Drinking water quality**		
Safe, %	76.5	(66.3, 84.4)
Unsafe, %	23.5	(15.6, 33.7)
**Household sanitation**		
Improved, %	37.8	(31.4, 44.8)
Unimproved, %	62.2	(55.2, 68.6)
**Children—micronutrient, inflammation, and malaria status (n = 710** ^d^**)**		
**Hemoglobin status**		
Hemoglobin (Hb; g/L), Mean, SD	9.72 (SD: 1.57)	(9.52, 9.92)
Anemia, %	76.3	(71.8, 80.4)
**Iron status**		
Ferritin, adjusted (μg/L), Median (IQR)	48.6	(26.1, 87.8)
Iron deficiency^†^, %	5.2	(3.3, 8.1)
Iron deficiency anemia, %	3.8	(2.5, 5.8)
**Vitamin A status**		
RBP, adjusted (μmol/l), Mean, SD	0.99 (SD: 0.35)	(0.96, 1.02)
Vitamin A deficiency, %	17.4	(13.9, 21.6)
**Inflammation**		
CRP (mg/L), Mean, SD	12.31 (SD: 18.38)	(10.31, 14.31)
AGP (g/L), Mean, SD	1.32 (SD: 0.51)	(1.26, 1.38)
Incubation, %	2.8	(1.6, 4.8)
Early convalescence, %	41.2	(36.3, 46.3)
Late convalescence, %	28.3	(24.4, 32.6)
None, %	27.7	(23.3, 32.6)
***Plasmodium falciparum*, %**	52.6	(46.0, 59.0)
**Non-pregnant women 15–49 years—micronutrient, inflammation, and malaria status (n = 871** ^e^**)**		
**Hemoglobin status**		
Hemoglobin (Hb; g/L), Mean, SD	12.0 (SD: 1.51)	(11.8, 12.1)
Anemia, %	44.8	(40.1, 49.5)
**Iron status**		
Ferritin, adjusted (μg/L), Median (IQR)	42.6	(24.2, 73.2)
Iron deficiency, adjusted, %	8.3	(6.2, 11.1)
Iron deficiency anemia, adjusted, %	6.1	(4.4, 8.6)
**Vitamin A status**		
RBP, adjusted (μmol/L), Mean, SD	1.45 (SD: 0.51)	(1.40, 1.51)
Vitamin A deficiency, adjusted, %	1.8	(1.1, 3.1)
Vitamin A insufficiency, adjusted, %	19.0	(15.6, 22.9)
**Folate & vitamin B12 status**		
Plasma folate (ηmol/L), Mean, SD	8.6 (SD: 5.1)	(8.1, 9.2)
Folate deficiency, %	79.2	(74.1, 83.5)
Plasma B12 (pmol/L), Mean, SD	556.2 (SD: 262.9)	(521.3, 591.0)
Vitamin B12 deficiency, %	0.5	(0.2, 1.4)
**Inflammation**		
CRP (mg/L), Mean, SD	3.85 (SD: 9.75)	(3.12, 4.57)
AGP (g/L), Mean, SD	0.78 (SD: 0.28)	(0.76, 0.80)
Incubation, %	7.3	(5.5, 9.6)
Early convalescence, %	9.2	(6.9, 12.1)
Late convalescence, %	7.2	(5.5, 9.2)
None, %	76.4	(72.7, 79.6)
***Plasmodium falciparum***, %	35.1	(30.1, 40.4)

^a^ Percentages weighted for unequal probability of selection.

^b^ CI = confidence interval, calculated taking into account the complex sampling design.

^c^ Clean cooking fuel = liquefied petroleum gas, biogas; Natural cooking fuels = wood, charcoal, coal/lignite, straw/shrubs/grass.

^d^ Sample size varies by analyte due to volume of blood collected from child.

Hb = 710; Malaria status = 723; ID-adjusted = 654; vitamin A deficiency = 654; CRP & AGP = 654.

^e^ Sample size varies slightly by analyte due to volume of blood collected from women.

Hb = 871; Malaria status = 833; ID = 774; vitamin A deficiency/insufficiency = 774; plasma folate = 776; plasma B^12^ = 768; CRP & AGP = 774.

### Children

Children <5 years old had an individual response rate of 87%, and 52.2% were female. Complete questionnaire and biomarker data were available for 654 children 6–59 months of age, and slightly higher sample sizes were obtained for hemoglobin (n = 710) and malaria (n = 723) status. As shown in Table 1, the mean hemoglobin concentration was less than 10 g/L, and more than three-quarters were anemic. Severe, moderate, and mild anemia was found in 5.4%, 45.8%, and, 25.2% of children, respectively. Median ferritin levels were 48.6 μg/L, resulting in 5.2% of children 6–59 months of age having iron deficiency; 3.8% had iron deficiency anemia, and 16.8% were vitamin A deficient after adjusting for inflammation. Overall, 72.6% (95% CI: 67.5, 77.1) of children had some type of inflammation. Current or recent malaria was found in more than one-half (52.6%) of the children. Anemia was more common in children from rural areas than those from urban areas, and in the North, South, and East regions than the West (Table 2). Children in households in the lowest four wealth quintiles have similar a similar prevalence of anemia, which are substantially higher than children in households in the wealthiest quintile. Anemia prevalence was higher in children with mothers with no formal education or only primary education than in children with mothers with a secondary or higher education. Children with positive malaria tests, recent diarrhea, and sub-clinical inflammation all have a significantly higher prevalence of anemia than children without these illnesses. Child age, sex, and malaria status show significant associations with iron deficiency. Malaria status was the only factor significantly associated with vitamin A deficiency, with a higher proportion (22.4%) of vitamin A deficiency in children with current or recent malaria compared to children without malaria (10.8%).

**Table 2:** Determinants of anemia, and iron and vitamin A deficiency in children 6–59 months of age, Sierra Leone 2013.

	Anemia (n = 710)			Iron deficiency (n = 654)			Vitamin A deficiency (n = 654)		
Characteristic	% ^a^, ^b^	(95% CI)^c^	P value ^d^	% ^a^, ^e^	(95% CI)^c^	P value ^d^	% ^f^	(95% CI)^c^	P value ^d^
Age Group (in months)									
6–11	88.1	(76.9, 94.3)	0.033	6.6	(3.0, 13.7)	0.085	15.8	(9.1, 26.0)	0.385
12–23	73.7	(64.4, 81.3)		8.8	(3.9, 18.8)		14.0	(9.4, 20.2)	
24–35	76.9	(69.4, 83.0)		6.4	(2.9, 13.4)		18.7	(10.8, 30.5)	
36–47	76.5	(68.6, 82.9)		3.6	(1.6, 8.2)		15.5	(9.9, 23.4)	
48–59	68.9	(60.0, 76.6)		—	—		24.1	(15.3, 35.8)	
Sex									
Male	78.3	(71.9, 83.6)	0.331	7.3	(3.9, 13.0)	0.050	15.4	(11.0, 21.1)	0.269
Female	74.5	(68.5, 79.7)		3.2	(1.8, 5.5)		19.4	(14.4, 25.5)	
Residence									
Urban	67.7	(60.9, 73.9)	<0.01	4.6	(2.2, 9.2)	0.659	15.6	(11.1, 21.4)	0.439
Rural	81.6	(75.1, 86.7)		5.6	(3.2, 9.6)		18.5	(13.7, 24.4)	
Region									
East	82.7	(75.7, 88.0)	<0.001	5.7	(2.5, 12.5)	0.385	16.0	(10.8, 23.0)	0.310
North	83.2	(73.7, 89.7)		7.2	(3.7, 13.6)		21.7	(16.0, 28.8)	
South	74.3	(65.8, 81.3)		2.4	(0.9, 6.6)		15.8	(9.4, 25.3)	
West	58.4	(51.8, 64.8)		4.8	(1.5, 14.1)		12.9	(7.0, 22.6)	
Mother's Education									
Never attended school	81.3	(75.7, 85.8)	<0.001	5.7	(3.2, 10.1)	0.660	17.5	(13.3, 22.6)	0.334
Primary school or less	81.0	(68.5, 89.3)		6.8	(2.9, 15.2)		20.4	(10.9, 34.9)	
Some secondary and above	63.7	(55.0, 71.7)		3.8	(1.3, 10.4)		11.8	(6.8, 19.8)	
Wealth Quintile									
Lowest	79.4	(69.7, 86.6)	<0.001	7.4	(3.1, 16.7)	0.400	12.2	(7.0, 20.4)	0.185
Second	83.1	(75.2, 88.8)		2.8	(0.9, 8.3)		23.6	(16.1, 33.3)	
Middle	81.0	(71.0, 88.1)		7.6	(3.4, 16.2)		22.9	(14.8, 33.6)	
Fourth	77.0	(69.3, 83.2)		3.8	(1.5, 9.3)		16.7	(10.0, 26.4)	
Highest	52.2	(43.2, 61.2)		4.3	(1.8, 9.8)		14.0	(6.7, 27.1)	
Malaria status ^g^									
Positive	88.1	(83.2, 91.8)	<0.001	3.0	(1.5, 5.8)	0.017	23.3	(18.4, 29.1)	<0.001
Negative	63.0	(5609, 68.8)		7.7	(4.5, 13.0)		10.8	(7.4, 15.4)	
Received deworming medication in past six months ^h^									
Yes	72.4	(66.8, 77.4)	0.478	4.7	(2.8, 7.9)	0.301	18.3	(13.9, 23.7)	0.212
No	77.4	(67.9, 84.7)		4.1	(1.1, 14.0)		15.3	(10.3, 22.0)	
Don’t know	67.5	(44.8, 84.2)		16.2	(3.3, 52.0)		31.8	(13.2, 58.8)	
Child had any type of diarrhea in the past 2 weeks									
Yes	80.9	(74.6, 86.0)	<0.05	5.2	(2.5, 10.7)	0.993	17.9	(11.8, 26.1)	0.877
No	74.2	(69.0, 78.8)		5.2	(2.9, 9.1)		17.2	(13.3, 22.1)	
Child had diarrhea with blood in the past 2 weeks									
Yes	88.5	(73.0, 95.7)	0.100	2.5	(0.3, 16.5)	0.428	16.2	(7.8, 30.7)	0.832
No	75.4	(70.4, 79.8)		5.4	(3.5, 8.4)		17.6	(13.9, 22.0)	
Child had a fever in the past 2 weeks									
Yes	76.3	(71.6, 80.5)	0.952	5.3	(3.1, 8.9)	0.914	18.3	(14.3, 23.1)	0.272
No	76.1	(67.0, 83.3)		5.0	(2.3, 10.5)		14.5	(9.6, 21.3)	
Child had lower respiratory infection									
Yes	79.2	(63.7, 89.1)	0.651	3.8	(0.8, 16.8)	0.654	15.1	(7.3, 28.7)	0.621
No	75.9	(70.9, 80.3)		5.5	(3.4, 8.6)		17.8	(14.4, 21.8)	
Inflammation									
Incubation	84.3	(55.4, 95.9)	<0.001	0	-	0.343	24.8	(8.8, 53.1)	0.125
Early convalescence	87.7	(80.7, 92.4)		6.6	(3.3, 12.6)		20.4	(15.2, 26.8)	
Late convalescence	77.0	(69.5, 83.1)		5.9	(3.1, 10.9)		18.6	(12.9, 26.1)	
None	58.6	(49.2, 67.3)		2.9	(1.4, 6.0)		10.9	(6.3, 18.2)	
Iron deficient ^e^									
Yes (ferritin <12 μg/L)	77.6	(41.2, 94.5)	0.934	—	—		8.5	(2.6, 24.5)	0.186
No (ferritin ≥12 μg/L)	76.4	(71.3, 80.8)		—	—		17.9	(14.2, 22.3)	
Vitamin A deficient									
Yes (RBP<0.7 μmol/L)	82.0	(72.7, 88.7)	0.221	2.6	(0.8, 8.7)	0.205	—	—	
No (RBP≥0.7 μmol/L)	75.7	(70.3, 80.4)		5.8	(3.6, 9.1)		—	—	

^a^ Percentages weighted for unequal probability of selection.

^b^ Anemia defined as hemoglobin <110 g/L adjusted for altitude.

^c^ CI = confidence interval, calculated taking into account the complex sampling design.

^d^ Chi-square p-value <0.05 indicates that the proportion in at least one subgroup is statistically significantly different from the values in the other subgroups

^e^ Iron deficiency defined as plasma ferritin <12 μg/L, values are adjusted for inflammation according to Thurnham [16].

^f^ Vitamin A deficiency defined as retinol binding protein <0.70 μmol/L.

^g^ Positive malaria status identified using rapid diagnostic tests during SLMS data collection

^h^ Includes only children 12–59 months of age (n = 597)

**Fig 1:**
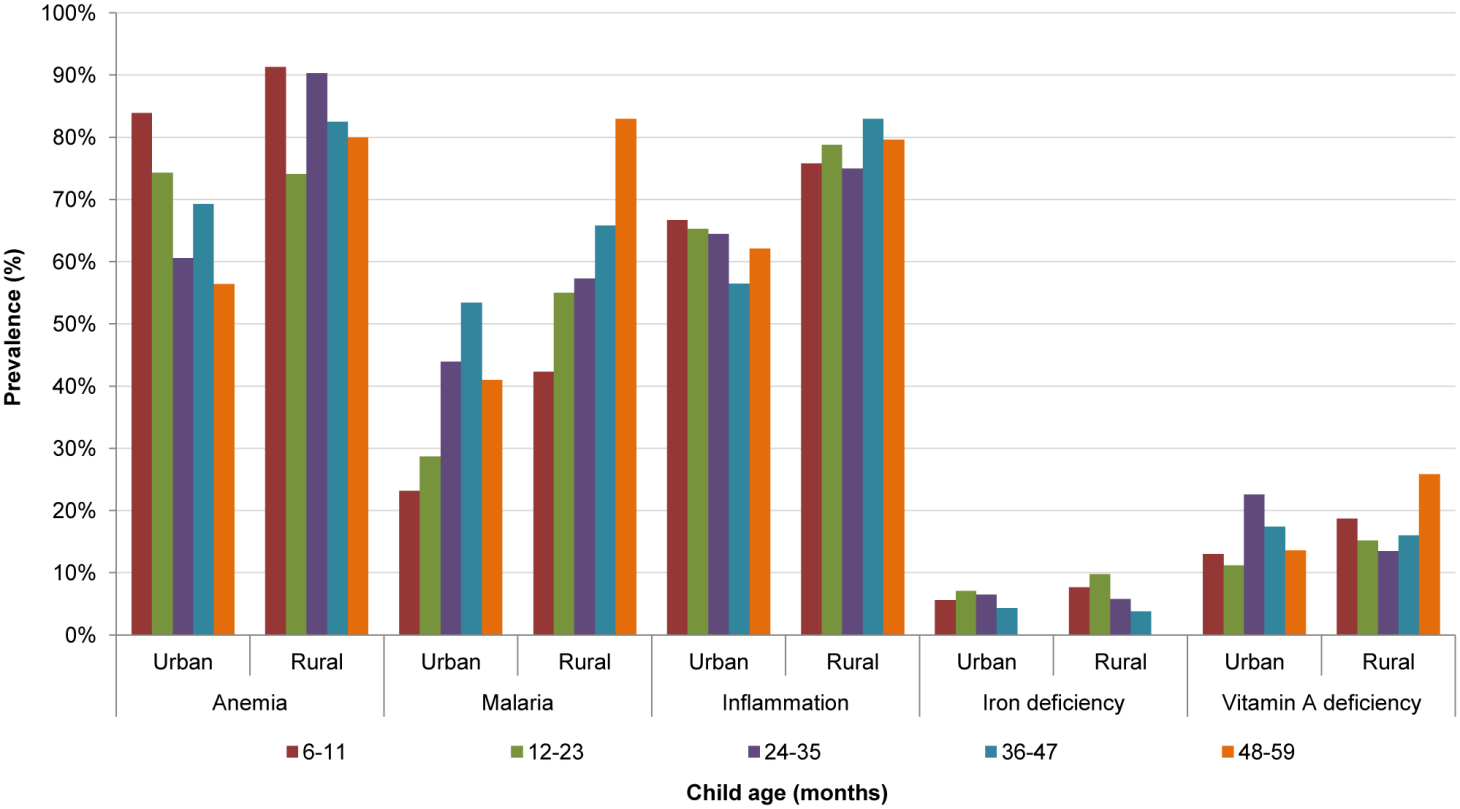
Anemia, malaria, inflammation, and iron and vitamin A deficiencies in children (6–59 months), by residence and age group, Sierra Leone 2013. Anemia, Hb <110g/L; malaria, positive for *Plasmodium*. *falciparum* using rapid diagnostic test; inflammation positive, CRP>5mg/L and/or AGP>1g/L; iron deficiency, ferritin <12μg/L adjusted for inflammation; vitamin A deficiency, RBP <0.7μmol/L adjusted for inflammation.

Fig 1 presents the prevalence of anemia, current or recent malaria, any inflammation, and iron and vitamin A deficiencies by age group and urban/rural residence. The prevalence of anemia was highest in children 6–11 months of age in both urban and rural areas. The anemia prevalence generally (but not consistently) declined by child age; in children 6–11 and 48–59 months of age the anemia prevalence ranged from 83.9% to 56.4% in urban areas and 91.3% to 80.0% in rural areas. In no sub-group was the prevalence <40%, the threshold to define a severe public health problem. Conversely, the prevalence of malaria increased by child age. In urban areas, the prevalence was highest (53.4%) among children 36–47 months of age; in rural areas, highest (83.0%) among children 48–59 months of age. The prevalence of inflammation and iron deficiency varied only slightly by age in urban and rural areas. For vitamin A deficiency, the highest prevalence was observed in urban children 24–35 months (22.6%) and rural children 48–59 months of age (25.8%).

When those factors showing statistically significant associations in bivariate analysis (see Table 2) were included in a logistic regression model with anemia as the outcome, only age, malaria status, and inflammation retain a statistically significantly association (Table 3). Children 6–11 months of age are much more likely to be anemic than older children. Children with current or recent malaria are also more likely to be anemic than children with recent malaria episode, and children with any stage of inflammation are more likely to be anemic than children without inflammation. The only factors significantly associated with iron deficiency in multivariate analysis were sex and malaria status, with male children and malaria-positive children more likely to be iron deficient. The population-attributable risks show that current or recent malaria accounted for 12.4% of anemia, and *any* inflammation accounted for 12.7% anemia. Nearly 75% of the anemia in children was not explained by the factors explored in our analysis.

**Table 3:** Multivariate logistic regression models for anemia and iron deficiency in children 6–59 months of age, Sierra Leone 2013.

	Odds Ratio	(95% CI) ^a^	P value
ANAEMIA MODEL (n = 557)			
Age Group (in months)			
6–11	5.1	(1.9, 14.1)	0.033
12–23	1.6	(0.74, 3.3)	
24–35	2.1	(0.92, 4.6)	
36–47	1.6	(0.89, 2.9)	
48–59	Referent		
Residence			
Rural	1.0	(0.51, 2.1)	0.994
Urban	Referent		
Region			
East	1.5	(0.54, 4.3)	0.714
North	1.1	(0.42, 3.1)	
South	1.0	(0.39, 2.5)	
West	Referent		
Mother's Education			
None	1.7	(0.98, 2.9)	0.141
Primary school or less	1.7	(0.75, 4.0)	
Some secondary and above	Referent		
Wealth Quintile			
Lowest	2.2	(0.84, 5.9)	0.510
Second	2.2	(0.80, 6.0)	
Middle	2.0	(0.68, 5.8)	
Fourth	2.1	(0.91, 4.6)	
Highest	Referent		
Malaria status ^b^			
Positive	3.3	(1.7, 6.5)	0.001
Negative	Referent		
Diarrhea in past 2 weeks			
Yes	1.1	(0.67, 1.7)	0.791
No	Referent		
Inflammation			
Incubation	2.0	(0.30, 14.0)	0.023
Early convalescence	2.5	(1.2, 5.1)	
Late convalescence	2.1	(1.2, 3.6)	
None	Referent		
IRON DEFICIENCY MODEL(n = 538)			
Age Group (in months) ^c^			
6–11	1.7	(0.55, 5.1)	0.519
12–23	2.3	(0.72, 7.2)	
24–35	1.8	(0.63, 5.4)	
36–47	Referent		
Sex			
Male	2.5	(1.1, 6.2)	0.039
Female	Referent		
Malaria status ^b^			
Positive	0.45	(0.22, 0.95)	0.036
Negative	Referent		

^a^ CI = confidence interval, adjusted for cluster sampling design.

^b^ Malaria status identified using rapid test kits testing for *Plasmodium falciparum*.

^c^ Children 48–59 months of age were not included because there were no iron deficiency in this age group.

### Women of reproductive age (both pregnant and non-pregnant)

Eighty-two percent of the randomly-selected non-pregnant women participated in the SLMS and had complete questionnaire and biomarker data. About one-half were anemic (Table 1). Severe, moderate, and mild anemia were found in 1.1%, 19.5%, and 24.2% of non-pregnant women, respectively. Median ferritin concentration was 42.6 μg/L and relatively few women had iron deficiency (8.3%) or iron deficiency anemia (6.1%);even fewer had vitamin A deficiency (2.1%). Although folate deficiency was common (79.2%), few women were affected by B_12_ deficiency (0.5%). Inflammation and current or recent malaria were found in approximately one-quarter and one-third of non-pregnant women, respectively.

Among pregnant women, the prevalence of anemia was 70.0% (95% CI: 62.9, 76.3); 2.3% (95% CI: 0.8, 6.1) had severe anemia, 39.6% (95% CI: 32.8, 46.7) had moderate anemia, and 28.2% (95% CI: 22.5, 34.6) had mild anemia. There was no statistically significant difference in the prevalence of anemia by age, residence, region, education, or household wealth. Malaria testing was positive in 28.6% (95% CI: 22.9, 35.0) of pregnant women, and the prevalence of malaria was statistically significantly higher in rural areas, 34.3%, compared to 20.6% in urban areas (p <0.05). Age, educational level, and household wealth were not statistically significantly associated with malaria.

In bivariate analyses, no statistically significant associations were found between demographic factors and anemia in non-pregnant women (Table 4). On the other hand, iron deficiency, malaria, and inflammation status were associated with anemia. Although there are statistically significant differences in the prevalence of anemia among the different inflammation groups, they are not consistent; women with both elevated CRP and AGP had a higher prevalence of anemia, but women with only CRP or AGP had lower prevalence rates of anemia than women without inflammation.

**Table 4:** Determinants of anemia, iron and folate deficiency in non-pregnant women 15–49 years of age, Sierra Leone 2013.

	Anemia (n = 871)			Iron deficiency (n = 774)			Folate deficiency (n = 766)		
Characteristic	% ^a^, ^b^	(95% CI)^c^	P value ^d^	% ^a^, ^e^	(95% CI)^c^	P value ^d^	% ^f^	(95% CI)^c^	P value ^d^
Age group (in years)									
15–19	46.0	(36.6, 55.7)	0.263	10.3	(5.6, 18.2)	0.595	84.2	(74.8, 90.5)	0.190
20–24	41.0	(32.2, 50.4)		10.2	(5.6, 17.7)		82.0	(73.5, 88.1)	
25–29	38.0	(30.0, 46.7)		5.5	(2.7, 10.9)		87.2	(79.3, 92.4)	
30–34	47.3	(37.0, 57.7)		9.8	(4.9, 18.7)		80.7	(69.6, 88.5)	
35–39	49.6	(37.3, 62.0)		8.7	(3.4, 20.6)		71.2	(59.2, 80.9)	
40–44	45.9	(32.7, 59.7)		5.8	(2.4, 13.2)		73.0	(59.4, 83.4)	
45–49	60.4	(44.4, 74.4)		3.2	(0.8, 12.6)		61.9	(39.5, 80.1)	
Residence									
Urban	42.9	(36.4, 49.6)	0.474	7.8	(5.0, 12.0)	0.719	82.3	(77.4, 86.3)	0.185
Rural	46.2	(39.7, 52.9)		8.7	(5.8, 12.9)		76.6	(68.2, 83.4)	
Region									
East	46.0	(34.3, 58.2)	0.481	9.9	(5.6, 17.1)	0.706	79.8	(61.2, 90.8)	0.180
North	48.5	(39.4, 57.7)		7.0	(3.7, 12.9)		75.7	(67.2, 82.6)	
South	43.5	(34.8, 52.5)		7.5	(4.5, 12.3)		88.2	(81.4, 92.8)	
West	39.2	(34.1, 44.7)		9.9	(5.8, 16.3)		74.9	(67.3, 81.2)	
Education									
Never attended school	46.2	(40.1, 52.5)	0.630	7.9	(5.4, 11.5)	0.384	78.1	(71.3, 83.7)	0.692
Primary school or less	45.6	(32.6, 59.2)		4.9	(2.2, 10.8)		78.0	(65.3, 87.0)	
Some secondary and above	41.9	(35.6, 48.5)		10.1	(5.9, 16.8)		81.3	(74.6, 86.5)	
Wealth Quintile									
Lowest	50.9	(41.6, 60.1)	0.485	4.6	(2.2, 9.2)	0.309	77.0	(62.0, 87.3)	0.948
Second	44.9	(35.5, 54.6)		8.6	(4.7, 15.3)		80.8	(72.5, 87.1)	
Middle	44.0	(33.5, 55.0)		9.0	(5.1, 15.6)		78.1	(65.0, 87.2)	
Fourth	44.5	(34.4, 55.1)		12.2	(6.7, 21.3)		80.5	(72.8, 86.5)	
Highest	38.5	(32.3, 45.1)		7.8	(4.5, 13.3)		78.1	(69.6, 84.7)	
Consumed iron tablets or syrup in past 6 months									
Yes	40.9	(33.3, 48.9)	0.23	11.3	(6.9, 18.0)	0.21	75.0	(66.5, 82.0)	0.136
No	46.5	(40.9, 52.2)		7.5	(5.0, 11.1)		80.6	(75.4, 84.9)	
Consumed foods rich in iron in past 24 hours									
Yes	45.5	(39.9, 51.1)	0.654	13.0	(9.6, 17.4)	0.770	80.3	(73.8, 85.5)	0.399
No	43.3	(35.5, 51.5)		12.2	(8.0, 18.1)		76.8	(69.8, 82.7)	
Iron status									
Deficient (<12 μg/L)	74.8	(61.8, 84.5)	<0.001	-	-	-	85.9	(73.1, 93.1)	0.177
Sufficient (≥12 μg/L)	40.9	(35.7, 46.2)		-	-		78.2	(73.1, 82.6)	
Consumed folic acid tablets in past 6 months									
Yes	40.5	(33.7, 47.7)	0.16	11.1	(7.4, 16.2)	0.09	77.7	(69.8, 84.0)	0.416
No	47.5	(40.9, 54.1)		7.0	(4.7, 10.4)		80.3	(75.5, 84.3)	
Consumed foods rich in folate in past 24 hours									
Yes	44.7	(39.8, 49.7)	0.968	12.1	(9.3, 15.6)	0.393	80.1	(75.0, 84.4)	0.216
No	44.9	(34.7, 55.6)		15.6	(8.6, 26.6)		74.1	(62.6, 83.0)	
Folate status									
Deficient (<10ηmol/L)	46.1	(40.6, 51.7)	0.218	9.1	(6.6, 12.5)	0.135	-	-	-
Sufficient (≥10ηmol/L)	39.1	(29.8, 49.4)		5.0	(2.2, 11.1)		-	-	
Consumed multi-vitamin tablets or syrup in past six months									
Yes	42.4	(34.9, 50.2)	0.47	11.2	(6.8, 17.9)	0.17	73.8	(65.1, 81.0)	0.046
No	45.3	(40.3, 50.5)		7.7	(5.5, 10.8)		80.4	(75.6, 84.5)	
Malaria status ^g^									
Positive	54.4	(46.5, 62.1)	<0.01	8.6	(4.9, 14.7)	0.97	81.4	(72.8, 87.8)	0.333
Negative	39.3	(33.8, 45.1)		8.5	(6.0, 11.9)		77.8	(72.6, 82.3)	
Inflammation									
Incubation	41.3	(27.0, 57.2)	<0.05	12.0	(4.9, 26.5)	0.55	77.9	(63.6, 87.7)	0.322
Early convalescence	58.5	(47.3, 68.9)		4.6	(1.5, 13.9)		84.5	(72.8, 91.7)	
Late convalescence	27.6	(15.6, 43.9)		9.9	(3.7, 24.1)		69.5	(52.1, 82.7)	
None	45.6	(40.2, 51.2)		8.3	(5.9, 11.4)		79.6	(73.9, 84.3)	
Vitamin A insufficiency									
Insufficient (<1.05 μmol/L)	51.5	(42.5, 60.4)	0.117	18.8	(11.4, 29.4)	0.093	86.4	(78.7, 91.6)	0.066
Sufficient (>1.05 μmol/L)	43.7	(38.4, 49.2)		11.3	(8.2, 15.3)		77.6	(71.5, 82.7)	

^a^ Percentages weighted for unequal probability of selection.

^b^ Anemia defined as hemoglobin <110 g/L adjusted for altitude.

^c^ CI = confidence interval, calculated taking into account the complex sampling design.

^d^ Chi-square p-value <0.05 indicates that the proportion in at least one subgroup is statistically significantly different from the values in the other subgroups

^e^ Iron deficiency defined as plasma ferritin <12 μg/L, values are adjusted for inflammation according to Thurnham [16].

^f^ Vitamin A deficiency defined as retinol binding protein <0.70 μmol/L.

^g^ Malaria status identified using rapid test kits testing for *Plasmodium falciparum*

No statistically significant associations were found between iron deficiency and the available demographic, nutritional, and health factors, most likely because the number of women with iron deficiency was relatively low. For folate deficiency, only consumption of multi-vitamin tablets or syrup in the past six months was significantly associated, with women consuming supplements displaying slightly less folate deficiency than those who did not take supplements.

Due to the small number of factors associated with iron and folate deficiencies and the very low prevalence of vitamin A deficiency, we only conducted multivariate analysis for anemia. Iron deficiency, malaria status, and inflammation were all highly significantly associated with anemia in the multivariate model (Table 5). As in the bivariate analysis, women with both elevated CRP and AGP were more likely to be anemic than women without inflammation, while those with either elevated CPR or AGP, but not both, were less likely to be anemic.

**Table 5:** Multivariate logistic regression model for anemia in non-pregnant women 15–49 years of age, Sierra Leone 2013.

	Odds Ratio	(95% CI) ^a^	P value
ANAEMIA MODEL (n = 741)			
Iron deficiency ^b^			
Yes	5.7	(2.8, 11.3)	<0.001
No	Referent		
Malaria status^c^			
Positive	2.0	(1.4, 3.0)	<0.001
Negative	Referent		
Inflammation			
Incubation	0.59	(0.26, 1.3)	<0.01
Early convalescence	1.9	(1.2, 3.0)	
Late convalescence	0.57	(0.22, 1.01)	
None	Referent		

^a^ CI = confidence interval, adjusted for cluster sampling design.

^b^ Iron deficiency defined as plasma ferritin <12 μg/L, values are adjusted for inflammation according to Thurnham [16].

^c^ Malaria status identified using rapid test kits testing for *Plasmodium falciparum*

The population attributable risk estimates of each risk factor's contribution to anemia demonstrate that iron deficiency causes 11.2% of anemia while current or recent malaria causes 13.8% of anemia. The status “any inflammation” accounted for no cases of anemia. As with children, the variables included in this analysis failed to explain about 75% of the anemia in non-pregnant women.

### Overlap of anemia, malaria, and inflammation

**Fig 2:**
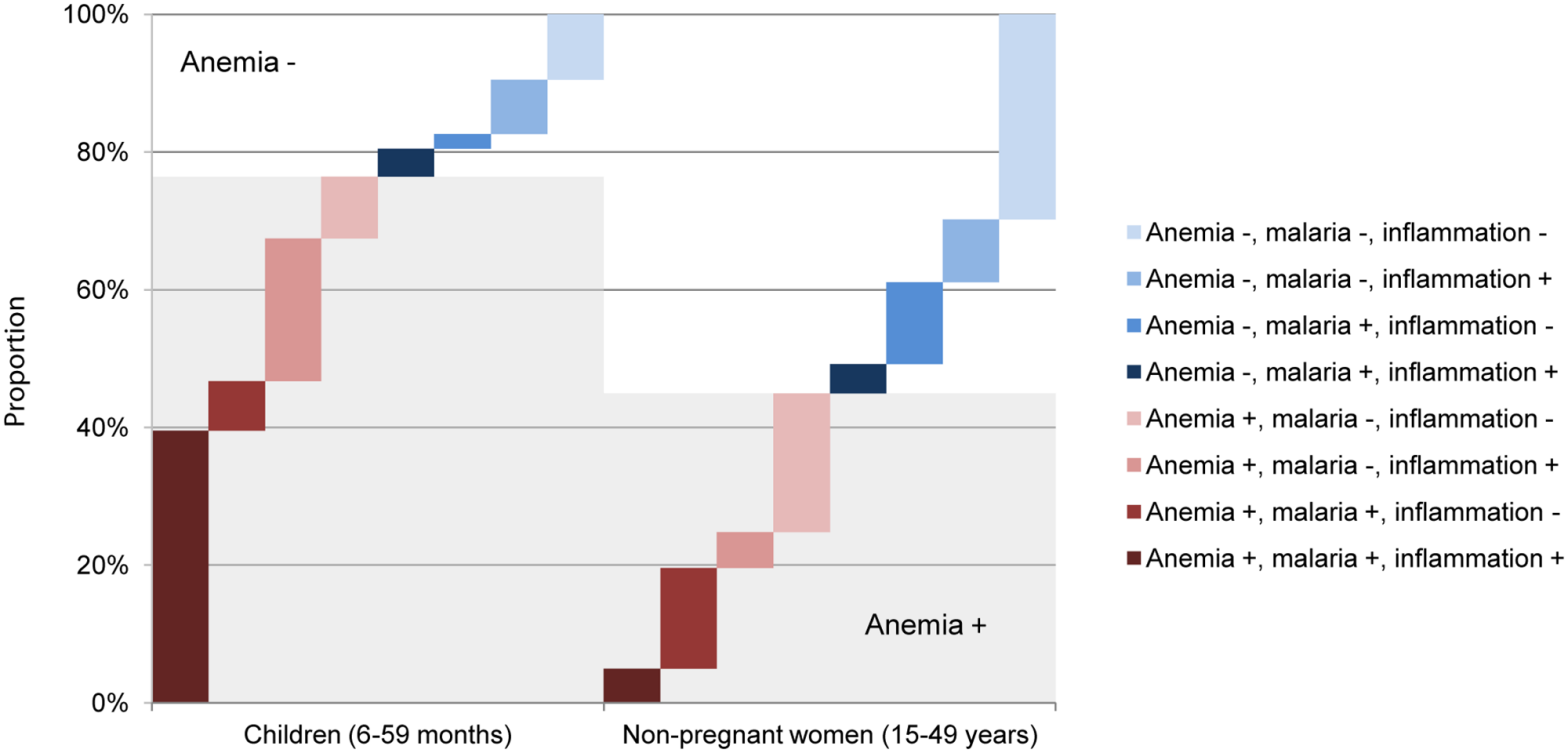
Overlap of anemia, malaria, and inflammation status in children (6–59 months) and non-pregnant women (15–49 years), Sierra Leone 2013. Anemia, Hb <110g/L in children and <120g/L in women; malaria, positive for *Plasmodium falciparum* using rapid diagnostic test; inflammation positive, CRP>5mg/L and/or AGP>1g/L.

Among children, malaria and inflammation were the only factors consistently associated with anemia. Nearly 40% of children had concurrent malaria and inflammation, and this co-morbidity was found in half of all anemic children (Fig 2). Anemia in the absence of malaria and/or inflammation was rare in children, and few non-anemic children possessed malaria and/or inflammation.

Concurrent malaria and inflammation was found in only 5.0% of all women, and present in 11.1% of anemic women. Malaria (without inflammation) affected similar proportions of anemic (14.6%) or non-anemic (11.9%) women, and inflammation (without malaria) affected a greater proportion of non-anemic (9.1%) than anemic (5.2%) women. Most notably, 20.1% of anemic women tested negative for inflammation and had a negative malaria rapid test.

## Discussion

### Anemia prevalence and determinants

The prevalence of anemia in Sierra Leonean children and women exceeds 40%, denoting a severe public health problem according to WHO classifications [15]. The anemia prevalence observed by the SLMS was similar to that found by Sierra Leone's 2013 Demographic Health Survey [25], which had a much larger sample size and was conducted six months prior to the SLMS. Moreover, the prevalence of anemia has not changed substantially since 2008 [26]. From 2008 to 2013, the prevalence of anemia in children only declined, 69.4% to 52.2%, amongst those in the highest wealth quintile, while the anemia prevalence of other wealth quintiles has remained at approximately 80%. Hence, only in the wealthiest Sierra Leoneans has anemia improved during that time period.

The high overlap of anemia and malaria in Sierra Leonean children has been previously observed elsewhere in Sub-Saharan Africa. A systematic review of observational studies from Sub-Saharan Africa found that malaria was more highly associated with anemia than measures of under-nutrition (i.e. stunting and wasting) [27]. As malaria can result in inflammation [28], and chronic inflammation can results in anemia [4], there was likely a compounding effect of both malaria and inflammation on anemia in children in Sierra Leone. As inflammation can occur independently of malaria in anemic children, anemia of chronic disease was also likely an independent cause of anemia in Sierra Leonean children.

Among the statistically significant risk factors for anemia in children, malaria and inflammation can be modified in order to prevent anemia. Although the risk factor age is non-modifiable, it does help target interventions to the age groups in greatest need. The peak of anemia prevalence among children 6–11 months of age, the typical period when complementary foods are introduced, may result from insufficient nutrient quality and/or quantity of complementary foods, increased exposure to infectious diseases, and/or reduced nutrient absorption. Because the standard infant and young child feeding indicators are calculated for relatively narrow age groups (e.g. children 6–8 months, 6–23 months, etc.), they were not examined in this analysis. The role of infant and young child feeding in micronutrient malnutrition should be investigated in greater detail.

Contrary to children, only a small proportion of women (5%) exhibited concurrent anemia, malaria, and inflammation. This suggests that malaria and inflammation are independent predictors of anemia in women. As 20.1% of anemic women did not display malaria or inflammation, there is likely other factors causing the anemia observed. Concurrent and independent malaria and inflammation was also found in non-anemic women, indicating that malaria and inflammation do not necessary result in anemia.

### Iron deficiency

Iron deficiency in Sierra Leone was relatively uncommon among children and women, in contrast to the general belief that iron deficiency accounts for approximately half of anemia in most populations [29]. Iron-deficiency was not associated with anemia in children and accounted for only a small proportion of anemia in women. Malaria was less prevalent in iron-deficient children, however, which may suggest that iron deficiency protects children from malaria parasitemia to some extent [30,31].

Serum ferritin was used as the indicator of iron deficiency. However, ferritin acts as an acute-phase reactant which increases in concentration during inflammation. The adjustment for inflammation that was applied prior to data interpretation may not have corrected for the entire effect [16], thus our measurement may have overestimated serum ferritin in the many children and women with inflammation. If true, such a misclassification bias would result in an underestimation of both the strength of association and the population attributable risk between iron deficiency and anemia would be underestimated. Nonetheless, the almost complete absence of any association in children, when many other surveys in populations with high inflammation prevalence rates of inflammation do show an association, casts doubt on the importance of iron deficiency as a cause of anemia in Sierra Leonean children. Though no comprehensive data of the mineral content of the drinking water in Sierra Leone could be found, high levels of blood iron in Sierra Leone may be a result of high iron content of soils, the majority is composed of are ferrallitic [32]. Ferrallitic soils are high in free iron, which may leach into drinking water. High levels of iron in drinking water have been associated with high iron stores in women in Cambodia [33] and Bangladesh [34], and may play a role in the low prevalence of iron deficiency in Sierra Leonean women and children.

### Vitamin A deficiency

The prevalence of vitamin A deficiency is considered a public health problem [20] in children, but not in women. Similar results have been found in Liberia [35] and Côte d’Ivoire [36], which suggests that vitamin A intake in children was sub-optimal whereas intake in women was sufficient. This finding is consistent with efforts in Sierra Leone to reduce vitamin A deficiency using twice-yearly vitamin A supplementation, which covers about 90% of children [37]. In 2013 the Ministry of Health and Sanitation introduced a novel strategy to increase the supplementation coverage through a routine maternal and child health “six-month contact point” [38]. This and other strategies should be used to maintain and expand the coverage vitamin A supplements. Vitamin A supplementation has been associated with lower levels of malaria [39], and our results suggest that vitamin A deficiency may make children more vulnerable to malaria. Thus, improvements in the vitamin A status of Sierra Leonean children may help to mediate the intensity of malaria.

In addition to biannual supplementation of vitamin A, increasing the regular dietary vitamin A intake in children is also warranted; promoting vitamin A-rich foods through food diversification and expanding the fortification of commercially-produced vegetable oil with vitamin A are options. However, as average daily consumption of vegetable oil was only about 4–5 grams in children and disproportionately reaches the less poor and urban households, adequately fortified vegetable oil may fail to reach a high proportion of children at greatest risk [40]. Biofortification approaches should also be considered; vitamin A-biofortified orange-fleshed sweet potatoes has been identified as a suitable approach to reduce vitamin A deficiency in Sierra Leone. Implementation of multiple programs to reduce vitamin A deficiency in children would be the most effective approach to ensuring sufficient and regular intake of vitamin A [41].

### Folate and B12 deficiencies

The SLMS found a very high prevalence of folate deficiency and a very low prevalence of vitamin B12 deficiency in women. While these deficiencies are often examined together due to their similar effects on erythropoiesis, the dietary sources of folate and B_12_ are quite different. These deficiencies were not, however, associated with low consumption of folate- and vitamin B_12_-rich foods. The SLMS found that in the 24-hours prior to being interviewed, 83.3% of women had consumed foods rich in folate (e.g. dark-leafy greens, legumes, fruits) and 71.5% of women had consumed foods rich in vitamin B_12_ (e.g. meat, offal, eggs, dairy products, seafood [42]). However, food frequency is a crude measure of dietary intake, and Sierra Leonean women's intake of folate and vitamin B_12_ requires further investigation. Hemoglobinopathies shorten the life span of red blood cells, increase erythropoiesis and the demand for folate. Persons known to have hemoglobinopathies should routinely receive folate supplements. The high level of hemoglobinopathies in this malaria-hyperendemic area can be contributing to the high level of folate deficiency found in this study.

Despite the high prevalence of folate deficiency in Sierra Leone, there was no association between folate deficiency and anemia. This finding is consistent with other, albeit sparse, evidence that suggest folate is *not* a major contributor of anemia in developing countries [43]. Although folate deficiency may not result in anemia in Sierra Leone, other adverse health outcomes of folate deficiency, such as neural tube defects, should be explored in view of the high deficiency prevalence. Of note, plasma folate is a short-term indicator of folate status, representing folate intake of the past 2–3 weeks [44]. Plasma folate does not capture the folate concentrations in erythrocytes, and plasma folate is not recommended by the WHO as an indicator to assess the risk of neural tube defects [45].

The prevalence of B_12_ deficiency reported (0.5%) was based on the WHO-endorsed deficiency cutoff of <150 pmol/L. Herbert [46] suggested a cut-off of <222 pmol/L as indicating the first stage of B_12_ depletion, or marginal vitamin B_12_ status. Using this alternative cutoff, 3.8% (95% CI: 2.2, 6.5) of women would be considered as having marginal and deficient vitamin B_12_ status. This finding illustrates that for the vast majority of women, vitamin B_12_ concentrations are at normal or higher levels. Vitamin B_12_ deficiency in Sierra Leone is considerably lower than that found in nationally-representative surveys in Côte d’Ivoire [36] and Cameroon [47], which found that 18% and 28% of non-pregnant women were deficient, respectively.

### Malaria

Sierra Leone's 2013 Malaria Indicator Survey (MIS), which was conducted approximately eight months prior to the SLMS, determined that 46.2% of children 6–59 months had a positive rapid diagnostic test for malaria [48]. The MIS unfortunately did not measure malaria prevalence in women of reproductive age. In children, the malaria prevalence of the MIS is slightly lower than the 52.6% observed by the SLMS. This may be due to the fact that the SLMS was conducted shortly after the rainy season when malaria is more prevalent, [25] than during the dry season when the MIS was conducted.

The government of Sierra Leone has identified malaria is one of the main public health problems affecting the country, and malaria-related illnesses account for 38% of all mortality in children < 5 years of age [49]. The government has undertaken considerable efforts at treatment, prevention, vector management, and other activities [49], and progress has been made in the recent past. To illustrate, the proportion of households possessing at least one insecticide-treated bednet increased from 37% to 62% from 2008 to 2013 [26,48]. Despite this increase, the coverage remains insufficient as only 17% of households had at least one insecticide-treated bednet for every two members [48].

Because our results indicate malaria is a key contributor to anemia, malaria prevention may contribute to anemia prevention. As of 2015, the Ministry of Health and Sanitation established an anemia working group to take a multi-sectoral approach to addressing anemia. The efforts of this working group will compliment the efforts of government and its stakeholders to treat and prevent malaria.

### Research and programmatic implications

Given the high prevalence of anemia and the relatively minor contribution of iron and vitamin A deficiencies, inflammation, and malaria, a thorough investigation of the etiology of anemia in Sierra Leone is needed before a comprehensive prevention and control strategy can be formulated. It is recommended that those potential contributing factors not measured by the SLMS be investigated, including hemoglobinopathies, such as sickle cell and thalassemia traits. Previous studies in Sierra Leone found that the prevalence of the sickle cell trait ranged from 22% [50] to 29% [51] and varied by ethnic group. The remaining blood pellets from SLMS blood specimens are available and should be assessed for hemoglobinopathies.

Following a thorough investigation of the etiology of anemia, large-scale programs to address the factors associated with anemia should be developed. Due the varying severity of anemia by residence, region, age, education, and wealth quintile, programs should be tailored and targeted at specific population groups.

## Conclusions

The prevalence of anemia is a severe public health problem for children and women in Sierra Leone, and based on our analysis, acute and chronic inflammation and malaria are the most consistent risk factors of anemia in both groups. In addition, vitamin A deficiency poses is a moderately severe public health problem in children, and high levels of folate deficiency were observed in women. Continued programmatic efforts should address the risk factors of anemia, reduce micronutrient deficiencies, and conduct further research to further describe the determinants of anemia.

## Supporting Information

S1 Appendix Household, child, and women English questionnaires used by the 2013 SLMS. (PDF)

## Abbreviations

AGP: a1-acid glycoprotein
CRP: C-reactive protein
MICS: Multiple-Indicator Cluster Survey
RBP: Retinol-binding protein
SLMS: Sierra Leone Micronutrient Survey
WHO: World Health Organization
