# Supplementary material for: Anemia, Micronutrient Deficiencies, and Malaria in Children and Women in Sierra Leone Prior to the Ebola Outbreak - Findings of a Cross-Sectional Study
Source: PLoS One. 2016 May 10;11(5):e0155031. doi: 10.1371/journal.pone.0155031 (PMC4862671; doi:10.1371/journal.pone.0155031)
Supplement: S1 Appendix — (PDF) [file pone.0155031.s001.pdf]

Affix HOUSEHOLD label here (starts with "H")	SIERRA LEONE NATIONAL MICRONUTRIENT SURVEY 2013

HOUSEHOLD QUESTIONNAIRE

1. Region East ....1<br>North ....2<br>South....................3<br>West....................4	2. Village/Place: ________________<br>3. Location of this cluster Urban .... 1<br>Rural 2
4. Cluster number .... ☐☐	5. Cluster control form HH number .... ☐☐
6. Name of head of household<br>________________	7. Team number.................... ☐
8. GPS Coordinates: North ___ ▪ ___ ___ ___ ___ ___	West ___ ___ ▪ ___ ___ ___ ___ ___

	Visit 1	Visit 2	Visit 3	
Date	__ __/__ __	__ __/__ __	__ __/__ __	9. Final visit ☐☐/☐☐/☐☐<br>Day Month Year
Interviewer no.	______	______	______	10. Interviewer no. .... ☐
Next visit: Date<br>Time	__ __/__ __<br>__ __ : __ __	__ __/__ __<br>__ __ : __ __	__ __/__ __<br>__ __ : __ __	11. Number of visits .... ☐
Result	___	___	___	12. Final result code .... ☐

FINAL RESULT CODES:

Completed 1
No household member or no competent respondent at home at time of visit 2
Entire household absent for long period or moved away ... 3
Refused 4
Dwelling vacant / Address not a dwelling 5
Dwelling destroyed 6
Dwelling not found 7
Other (specify) ________________ 8

Note: Questions 13 and 15 should be filled in following the completion of the household roster. Questions 14 and 16 to be filled in after the completion of the individual questionnaires.

13. Number of children 0-59 months .... ☐☐	(circle #)<br>14. Number of children with data 0 1
15. Number of NPW .... ☐☐	(circle #)<br>16. Number of NPW with data 0 1 2

Hello. We are working with the Ministry of Health and Sanitation in Sierra Leone. We are conducting a national nutrition survey to better understand various types of nutritional problems such as anemia, and vitamin and mineral deficiencies in women and children. This information will help the government to plan for better health in the future. We would very much appreciate your household's participation in this survey. The survey usually takes about 45 minutes to 1 hour to complete, and includes answering questions and a visit to another place to take a small blood sample from the women and children in the household. Whatever information you provide will be kept strictly confidential and will not be shown to other persons.

Participation in this survey is voluntary, and if we should come to any question you do not want to answer, just let me know and I will go on to the next question; or you can stop the interview at any time. However, we hope that you will participate in this survey since your views are important. After these questions to you, I will speak with some of the women in your household and the women who take care of the children 0-59 months.

17. May I start now?

- ☐ YES, PERMISSION IS GIVEN -> BEGIN THE INTERVIEW.
- ☐ NO, PERMISSION IS NOT GIVEN -> COMPLETE THIS COVER PAGE. DISCUSS RESULT WITH TEAM LEADER.

First, I would like to ask you some general questions about the people who live in this household. Please tell me the name of each person who usually lives here, starting with the head of the household. *List the head of the household in line 01. List all household members and their sex. Then ask:* **Are there any others who live here, even if they are not at home now?** *If yes, complete listing for questions 16-19. Then, ask questions starting with 18 for each person at a time. Use an additional questionnaire if all rows in the household listing form have been used.*

Household Roster

							Women age 15-49	Pregnant	Children age < 5 years	Children < 5 years
18. Line No		19. **Is** (name) **male or female?**		20. **What is** (name)'s **date of birth?** 99 DK	9999 DK	21. **How old is** (name)? *Record in completed years. If age is 95 or above, record '95* *99 DK*	22. *Circle line no. if woman is age 15-49 years*	23. *Circle line no. if pregnant*	24. *Circle line no. if child is age 0-59 months*	25. **Who is the mother or primary caretaker of this child?** *Record line no. of mother or caregiver*
Line	Name	M	F	Month	Year	Age	15-49		0-59	caregiver
01	Household Head	1	2	__ __	__ __ __ __	__ __	01	01	01	__ __
02		1	2	__ __	__ __ __ __	__ __	02	02	02	__ __
03		1	2	__ __	__ __ __ __	__ __	03	03	03	__ __
04		1	2	__ __	__ __ __ __	__ __	04	04	04	__ __
05		1	2	__ __	__ __ __ __	__ __	05	05	05	__ __
06		1	2	__ __	__ __ __ __	__ __	06	06	06	__ __
07		1	2	__ __	__ __ __ __	__ __	07	07	07	__ __
08		1	2	__ __	__ __ __ __	__ __	08	08	08	__ __
09		1	2	__ __	__ __ __ __	__ __	09	09	09	__ __
10		1	2	__ __	__ __ __ __	__ __	10	10	10	__ __
11		1	2	__ __	__ __ __ __	__ __	11	11	11	__ __
12		1	2	__ __	__ __ __ __	__ __	12	12	12	__ __
13		1	2	__ __	__ __ __ __	__ __	13	13	13	__ __
14		1	2	__ __	__ __ __ __	__ __	14	14	14	__ __
15		1	2	__ __	__ __ __ __	__ __	15	15	15	__ __
16		1	2	__ __	__ __ __ __	__ __	16	16	16	__ __
17		1	2	__ __	__ __ __ __	__ __	17	17	17	__ __
18		1	2	__ __	__ __ __ __	__ __	18	18	18	__ __
19		1	2	__ __	__ __ __ __	__ __	19	19	19	__ __
20		1	2	__ __	__ __ __ __	__ __	20	20	20	__ __

Probe for additional household members. Probe especially for any infants or small children not listed, and others who may not be members of the family (such as servants, friends) but who usually live in the household. Insert names of additional members in the household list and complete form accordingly.

A household is defined a person or a group of persons, related or unrelated, who live together and share a common source of food and livelihood, and recognize one person as a head

To randomly select women and children from the household, use the information in this household roster to complete the following page. For each individual selected, prepare the first and last page of the questionnaire, including the labels. You should now have a separate questionnaire for each selected woman and child < 5 years in the household.

To randomly select women and children from the households, all eligible women and children from the household roster must be listed according to the instructions below. Follow the four steps in order.

Pregnant women

- List all eligible pregnant women (Q23) below.

1

Age	Name	Line number on HH roster

→ Prepare questionnaires for ALL pregnant women.

Non-pregnant women:

- List all eligible non-pregnant women (15-49 yrs) in order of increasing age.
- Record age in years (Q21), name, and line number (Q18) below.
- Consult Kish table and record line number of randomly selected women in Box A.

2

Age	Name	Line number on HH roster

→ **Box A**

Children and mothers or caregivers:

- List all eligible children (< 5 years) in order of increasing age.
- Record age in years (Q21), name, line number (Q18), and mother's or caregiver's number.
- Consult Kish table and record randomly selected child in box B.
- Fill in the line number of the corresponding mother or caregiver in box C.

3

Age	Name	Child's line number on HH roster	Mother's or caregiver's line number on HH roster

→ **Box B** **Box C**

4 →

Prepare questionnaires for:

- ALL pregnant women (step 1).
- ALL non-pregnant women and children in boxes A, B, and C (steps 2 & 3).

If the line numbers in boxes A and C are the same, prepare only one questionnaire.

If the woman listed in box C is pregnant, only prepare one questionnaire for this woman.

Now I would like to ask you about some basic questions about the household head

26. **What is the religion of the head of this household?**	Christian .... 1<br>Muslim .... 2<br>Traditional .... 3<br>No religion .... 7<br>Other religion (specify) ________ . 8<br>Don't know 9	
27. **What is the first language of the head of this household?**	Mende .... 1<br>Temne .... 2<br>Limba .... 3<br>Creole .... 4<br>Madingo .... 5<br>Loko .... 6<br>Sherbro .... 7<br>Kono .... 8<br>Other (specify) ________ 88<br>Don't know 99	
28. **Has the head of this household ever attended school or preschool?**	Yes .... 1<br>No .... 2<br>Don't know 9	-> Next Q<br>-> Q30<br>-> Q30

29. **What is the highest level of school <u>attended</u> by the head of this household?**

How many years at this level did he/she <u>complete</u>?

	Circle code	Circle # years completed	DK
Kindergarten	0	0 1 2 3	9
Primary	1	0 1 2 3 4 5 6 7	9
JSS - Junior Secondary ..	2	0 1 2 3	9
SSS - Senior Secondary .	3	0 1 2 3	9
Vocational/ commercial/ nursing/ technical/ teaching	4	0 1 2 3	9
Tertiary/college/univ	5	0 1 2 3 4 5 6	9
Don't know	9		

Now I would like to ask you about some basic questions about the household's characteristics

30. **How many rooms in this household are used for sleeping?**	Number of rooms .... ☐☐	
31. Main material of dwelling floor<br><br>*<u>Record only 1 observation</u>*	Natural floor<br>Earth / Sand .... 11<br>Dung .... 12<br>Stone .... 13<br>Rudimentary floor<br>Wood planks .... 21<br>Palm / Bamboo .... 22<br>Finished floor<br>Parquet or polished wood .... 31<br>Vinyl or asphalt strips .... 32<br>Ceramic tiles .... 33<br>Cement .... 34<br>Carpet .... 35<br><br>Other (specify) ________ 88	

32. Main material of the roof<br><br>*Record only 1 observation*	Natural roofing<br>No Roof .... 11<br>Thatch / Palm leaf .... 12<br>Sod .... 13<br>Rudimentary Roofing<br>Rustic mat .... 21<br>Palm / Bamboo .... 22<br>Wood planks .... 23<br>Cardboard .... 24<br>Finished roofing<br>Metal / Zinc........ 31<br>Wood .... 32<br>Calamine / Cement fibre .... 33<br>Ceramic tiles .... 34<br>Cement .... 35<br>Roofing shingles .... 36<br>Other (specify) ______________ 88	
33. Main material of the exterior walls.<br><br>*Record only 1 observation*	Natural walls<br>No walls .... 11<br>Cane / Palm / Trunks .... 12<br>Dirt .... 13<br>Rudimentary walls<br>Bamboo with mud .... 21<br>Stone with mud .... 22<br>Uncovered mud brick .... 23<br>Plywood .... 24<br>Cardboard .... 25<br>Reused wood .... 26<br>Metal / Zinc .... 27<br>Finished walls<br>Cement .... 31<br>Stone with lime / cement .... 32<br>Bricks .... 33<br>Cement blocks .... 34<br>Covered mud brick .... 35<br>Wood planks / shingles .... 36<br>Other (specify) ______________ .88	
34. **What type of fuel does your household mainly use for cooking?**<br><br>*Record only 1 response*	Electricity .... 1<br>Liquefied Petroleum Gas (LPG)........ 2<br>Natural gas .... 3<br>Biogas .... 4<br>Kerosene .... 5<br>Coal / Lignite .... 6<br>Charcoal........ 7<br>Wood........ 8<br>Straw / shrubs / grass .... 9<br>Animal dung .... 10<br>Agricultural crop residue .... 11<br>No food cooked in household .... 77<br>Other (specify: ______________) .... 88<br>Don't know 99	

Now I would like to ask you some questions about things people in your household may own and things you may use at home.

35. **Does your household have _____?**<br><br>*Ask about each item separately.*	Yes No<br>A. **Electricity?** .... 1 2<br>B. **A television?** .... 1 2<br>C. **A refrigerator?** .... 1 2<br>D. **Non-mobile telephone?** .... 1 2<br>E. **A radio?** 1 2	
36. **Does anyone in your household own a _____?**<br><br>*Ask about each item separately.*	Yes No<br>A. **Watch?** .... 1 2<br>B. **Mobile phone?** .... 1 2<br>C. **Bicycle?** .... 1 2<br>D. **Motorcycle?** .... 1 2<br>E. **Car / Truck?** .... 1 2<br>F. **Canoe?** .... 1 2<br>G. **Boat with motor?** .... 1 2<br>H. **Wheel barrow?** .... 1 2<br>I. **Sprayer?** .... 1 2<br>J. **Electric rice cutter?** .... 1 2<br>K. **Sickle / Knife?** .... 1 2<br>L. **Shovel / Hoe?** 1 2	
37. **Do you or someone living in this household own this dwelling?**	Yes .... 1<br>No .... 2<br>Don't know 9	
38. **Does any member of this household own any land that can be used for agriculture?**	Yes .... 1<br>No .... 2<br>Don't know 9	-> Next Q<br>-> Q40<br>-> Q40
39. **If yes, how much agricultural land do members of this household own?**<br><br>*Fill in agricultural land size for 1 category only.*	A. Plot .... ☐☐☐<br>B. Town lot.......................... ☐☐☐<br>C. Acres .... ☐☐☐<br><br>Don't know 999	
40. **Does this household own any livestock, herds, other farm animals, or poultry?**	Yes .... 1<br>No .... 2<br>Don't know 9	-> Next Q<br>-> Q42<br>-> Q42
41. **How many of the following animals does this household own?**<br><br>*Ask about each item separately.*<br><br>*If none, enter '00'*<br>*If more than 95, enter '95'*<br>*If unknown, enter '99'*	A. **Cattle, cows, bulls**................. ☐☐<br>B. **Horses, donkeys, mules** ..... ☐☐<br>C. **Goats** .... ☐☐<br>D. **Sheep** .... ☐☐<br>E. **Rabbits** .... ☐☐<br>F. **Pigs** .... ☐☐<br>G. **Fowl** (Chickens, geese, ducks, turkeys) ☐☐<br>H. **Rodents to breed**.................... ☐☐<br>I. **Birds to sell**............................ ☐☐<br>J. **Bees** (Number of Hives) .... ☐☐<br>K. Other (______________) ...... ☐☐	

Now I would like to ask you about drinking water and sanitation in your household.

42. **What is the main source of drinking water for members of your household?**<br><br>*Record only 1 response.*	Piped water<br>Piped into dwelling .... 11<br>Piped into compound, yard or plot .... 12<br>Piped to neighbour .... 13<br>Public tap / standpipe .... 14<br>Tube well or borehole or handpump .... 21<br>Dug well<br>Protected well.. 31<br>Unprotected well .... 32<br>Water from spring<br>Protected spring .... 41<br>Unprotected spring................................ 42<br>Rainwater collection .... 51<br>Tanker-truck .... 61<br>Cart with small tank or drum .... 71<br>Surface water (river, stream, dam, lake, pond, canal, irrigation channel)............. 81<br>Bottled water .... 91<br>Other (specify)______________________ 88<br>Don't know 99	
43. **Do you do anything at home to the water to make it safer to drink?**	Yes .... 1<br>No.. 2<br>Don't know 9	-> Next Q<br>-> Q45<br>-> Q45
44. **What do you usually do to make the water safer to drink?**<br><br>*Probe*: **Anything else?**<br><br>*Record all responses mentioned.*	Boil .... A<br>Add bleach or chlorine .... B<br>Strain it through a cloth .... C<br>Use water filter (ceramic, sand, composite, etc.) .... D<br>Solar disinfection....................................... E<br>Let it stand and settle .... F<br><br>Other (specify)______________________ Y<br>Don't know .... Z	
45. **What is the main source of water used for washing utensils?**<br><br>*Record only 1 response.*	Piped water<br>Piped into dwelling .... 11<br>Piped into compound, yard or plot .... 12<br>Piped to neighbour .... 13<br>Public tap / standpipe .... 14<br>Tube well or borehole .... 21<br>Dug well<br>Protected well.. 31<br>Unprotected well .... 32<br>Water from spring<br>Protected spring .... 41<br>Unprotected spring................................ 42<br>Rainwater collection .... 51<br>Tanker-truck .... 61<br>Cart with small tank or drum .... 71<br>Surface water (river, stream, dam, lake, pond, canal, irrigation channel)............. 81<br>Bottled water .... 91<br>Other (specify)______________________ 88<br>Don't know 99	

46. **What kind of toilet facility do members of your household usually use?**<br><br>*If "flush" or "pour flush", probe:*<br>**Where does it flush to?**<br><br>*If necessary, ask permission to observe the facility.*<br><br>*Record only 1 response.*	Flush / Pour flush<br>Flush to piped sewer system .... 11<br>Flush to septic tank .... 12<br>Flush to pit (latrine) .... 13<br>Flush to somewhere else .... 14<br>Flush to unknown place/ not sure / don't know where .... 15<br>Pit latrine<br>Ventilated Improved Pit latrine (VIP) ..... 21<br>Pit latrine with slab .... 22<br>Pit latrine without slab / Open pit .... 23<br><br>Composting toilet .... 31<br>Bucket .... 41<br>Hanging toilet, Hanging latrine .... 51<br>No facility, Bush, Field .... 61<br>Other (*specify*)________ 88<br>Don't know 99	 <br><br><br><br><br><br><br><br><br><br><br><br>-> Q48<br>-> Q48<br>-> Q48
47. **Do you share this facility with others who are not members of your household?**	Yes .... 1<br>No .... 2<br>Don't know 9	
48. **Please show me where members of your household most often wash their hands.**	Observed (Sink or fixed basin) .... 1<br>Observed (Anywhere around dwelling) .... 2<br><br>Not observed<br>Not in dwelling / plot / yard .... 3<br>No permission to see .... 4<br>Other reason (*specify*)________ 8	 <br><br><br>-> Q51<br>-> Q51<br>-> Q51
49. *Observe presence of water at the specific place for handwashing. Verify by checking the tap/pump, or basin, bucket, water container or similar objects for presence of water.*	Water is available .... 1<br><br>Water is not available 2	
50. *Record if soap or detergent is present at the specific place for handwashing.*<br><br>*Circle 1 for Yes for each type of soap seen.*<br><br>*Skip to Q52 if any soap or detergent code (A, B, or C) is YES. If D and E is circled YES, continue with next question.*	Yes No<br>A. Bar soap .... 1 2<br>B. Detergent .... 1 2<br>(Powder / Liquid / Paste)<br>C. Liquid soap .... 1 2<br>D. Ash / Mud / Sand .... 1 2<br>E. None 1	 -> Q52<br>-> Q52<br><br>-> Q52<br>-> Next Q<br>-> Next Q
51. **Do you have any soap or detergent in your household for washing hands?**<br><br>If Yes:<br>**Can you please show it to me?**<br>*Circle Yes for each type of soap seen.*<br><br>If No, circle Yes for E.	Yes No<br>A. Bar soap .... 1 2<br>B. Detergent .... 1 2<br>(Powder / Liquid / Paste)<br>C. Liquid soap .... 1 2<br>D. Ash / Mud / Sand .... 1 2<br>E. None 1	

Now I would like to ask you some questions about the salt most commonly used in this household.

52. **Do you have salt in your house now?**	Yes .... 1<br>No .... 2<br>Don't know 9	-> Next Q<br>-> Q56<br>-> Q56
53. **May I have a small sample of the salt that you use for cooking?**<br><br>Collect approximately 20-30 grams of salt to be used for quantitative testing at central laboratory.	Yes .... 1<br><br>No 2	-> Collect Salt<br>-> Q56

54. SALT SPECIMEN COLLECTED?	Yes .... 1<br>No 2	
55. Does salt container show that it is iodized?<br>*Observe the package that salt is in*	Yes, original package says iodized .... 1<br>Original package not mention iodization ... 2<br>Undetermined, not in original package ..... 3<br>Undetermined for other reason 8	

56. **How many times per day, week, or month do you usually purchase vegetable oil?**<br>*Fill in number of times for 1 time period only.*	Number of times a:<br>A. ☐ times per Day<br>B. ☐☐ times per Week<br>C. ☐☐ times per Month<br>I don't use it .... 00<br>Don't know / not sure 99	 <br><br><br><br>->Q58<br>->Q58
57. **What quantity is usually obtained whenever vegetable oil is bought?**<br>*Fill in quantity for 1 unit of measure only*	A. Pint(s) .... ☐☐<br>B. Rubber(s) .... ☐☐<br>C. Gallon(s) .... ☐☐<br>D. Don't know / not sure 99	
58. **What type of food products made with wheat flour do you eat <u>most often</u> in this household?**	Bread .... 1<br>Pan cakes .... 2<br>Doughnuts .... 3<br>Other (specify ____________) . 8<br>Unknown 9	-> Next Q<br>-> END<br>-> END<br>-> END<br>-> END
59. **What type of bread do you eat most often in this household?**	Factory white bread .... 1<br>Factory brown bread .... 2<br>Other bread from bakery or factory .... 3<br>Home-made .... 4<br>Other (specify ____________) . 8<br>Unknown 9	
60. **How many times per day, week, or month do you usually purchase bread?**<br>*Fill in number of times for only 1 time period.*	Number of times a:<br>A. ☐ times per Day<br>B. ☐☐ times per Week<br>C. ☐☐ times per Month<br>D. Don't know / not sure 99	
61. **What quantity is usually obtained whenever bread is bought?**<br>*Fill in number of loaves for either full-size loaves, medium loaves, or small loaves, if BOTH is bought then fill BOTH*	A. Number of full-size loaves .... ☐☐<br>B. Number of medium loaves .... ☐☐<br>C. Number of small loaves (baguettes) . ☐☐<br>D. Don't know / not sure 99	

Comments about data collection at this household:

The form was reviewed by: ____ Date: ________________

Team leader's signature

Data entry clerk name: __________________________________ Data entry clerk code number: ☐

Affix HOUSEHOLD label here (starts with "H")	**CHILD QUESTIONNAIRE**<br>**SIERRA LEONE NATIONAL MICRONUTRIENT SURVEY 2013**	Affix CHILD label here (starts with "C")

1. Cluster number .... ☐☐	2. Cluster control form HH number .. ☐☐
3. Name of this child: ____________________________	4. Child number................................ ☐☐
5. Interviewer number.. ☐☐	
6. Date of data collection ☐☐ / ☐☐ / ☐☐<br>Day Month Year	7. Child's mother's woman number:... ☐☐
8. Final result of child data collection .... ☐	
FINAL RESULT CODES:<br>Completed interview and accepted participation in blood collection .... 1<br>Completed interview and refused participation in blood collection 2	Refused interview and all data collection .... 3<br>Child not at home at time of visit .... 4<br>Other (specify__________________________)...8

Child questionnaire should be administered to the child's caregiver as identified by the household roster.<br><br>Repeat greeting if not already read to this respondent.<br><br>**We are working with the Ministry of Health and Sanitation in Sierra Leone. We are conducting a national nutrition survey to better understand the various nutritional deficiencies, such as such as anemia, vitamins and minerals in women and children. This information will help the government to plan for better health in the future.**	If greeting at the beginning of the household questionnaire has already been read to this woman, then read the following:<br>**Now I would like to talk to you more about (***child's name***)'s health and other topics. When I ask about (***child's name***), please think only of this child and answer only about this child. Try not to mix up other children in the household. All the information we obtain will remain strictly confidential and your answers will never be shared with anyone other than our project team.**<br><br>**The survey usually takes about 20 minutes to complete, and includes answering questions and a visit to (**location of laboratory site**) to take a small blood sample of (***child's name***).**
**9. May I start now?**<br>☐ YES, PERMISSION IS GIVEN ->BEGIN THE INTERVIEW.<br>☐ NO, PERMISSION IS NOT GIVEN ->COMPLETE THIS COVER PAGE. DISCUSS THIS RESULT WITH YOUR TEAM LEADER.	

Now I would like to ask you some basic questions about (NAME).

10. **Is (NAME) a boy or girl?**	Male ....1<br>Female 2	
11. **What is (NAME)'s date of birth?**<br><br>*Copy date of birth from document(or child health card) if available, or probe:*<br><br>**What month and year was (NAME) born?**	D. Day....................... ☐☐<br>(enter '99' if unknown)<br><br>M. Month................... ☐☐<br><br>Y. Year...................... ☐☐	
12. **How old is (NAME) in completed months?**<br>*If necessary, use local calendar to derive age.*<br>*Record '99' if unknown.*<br>*Compare date of birth above and stated age; correct one if necessary.*	Age (in months).......... ☐☐<br><br>(in completed months)<br>(enter '99' if unknown)	
13. **Was (NAME) weighed at birth?**	Yes....................................... 1<br>No .... 2<br>Don't know 9	-> Next Q<br>->Q15<br>->Q15

14. **How much did (name) weigh?**<br>*Record weight from health card, if available*	A. From card<br>(kg) ....☐.☐☐☐<br>B. From recall<br>(kg) ....☐.☐☐☐<br>Don't know ....9.999 in B	
15. **Did you give birth to this child?**	Yes .... 1<br>if "Yes", please note her line number on the household roster (Q18; HH questionnaire)<br>A. Mother number ......☐☐<br>No 2	-> Q18<br><br>-> Next Q
16. **Is the woman who gave birth to (NAME) alive?**	Yes...................................... 1<br>No .... 2<br>Don't know 9	-> Next Q<br>-> Q18<br>-> Q18
17. **Does (NAME's) biological mother live in this household?**	Yes .... 1<br>if "Yes", please note her line number on the household roster (Q18; HH questionnaire)<br>A. Mother number ......☐☐<br>No 2	
18. **Is (NAME's) father alive?**	Yes...................................... 1<br>No .... 2<br>Don't know 9	

Now I would like to ask you about illnesses (NAME) may have had in the past 2 weeks. Please keep in mind only this time period; do not include any illnesses (NAME) had before 2 week ago.

19. **At any time in the last 2 weeks, has (NAME) had diarrhoea?**<br>*Diarrhoea = watery stool at least three times per day*	Yes... 1<br>No .... 2<br>Don't know 9	-> Next Q<br>-> Q21<br>-> Q21
20. **Was there any blood in the stools?**	Yes.. 1<br>No .... 2<br>Don't know 9	
21. **At any time in the last 2 weeks, has (NAME) been ill with a fever?**	Yes... 1<br>No .... 2<br>Don't know 9	-> Next Q<br>-> Q24
22. **At any time during this illness with fever, did (name) have blood taken from his/her finger or heel for malaria testing?**	Yes... 1<br>No .... 2<br>Don't know 9	->Next Q<br>-> Q24<br>-> Q24
23. **Did that test show that (name) had malaria?**	Yes... 1<br>No .... 2<br>Don't know 9	
24. **At any time in the last 2 weeks, has (NAME) had an illness with a cough?**	Yes... 1<br>No .... 2<br>Don't know 9	->Next Q<br>-> Q27<br>->Q27
25. **When (NAME) had an illness with a cough, did he/she breathe faster than usual with shallow, rapid breaths or have difficulty breathing?**	Yes... 1<br>No .... 2<br>Don't know 9	-> Next Q<br>-> Q27<br>-> Q27
26. **Was the fast or difficult breathing due to a problem in the chest or a blocked or runny nose?**	Problem in chest only .... 1<br>Blocked or runny nose only ..... 2<br>Both .... 3<br>Other (specify) ___________ ..... 8<br>Don't know 9	

NOTE: Dietary questions (questions 27-37) are to be asked ONLY about children 0 – 23 months of age. Check the child's date of birth and age above. If the child is 24 months of age or older, skip to question 38.

Now I will ask you questions about (NAME)'s diet. Please answer only for (NAME). Do not confuse (NAME) with other young children in the household.

27. **Has (NAME) ever been breastfed?**<br>*Include giving breastmilk by spoon or bottle or breastfeeding by other women.*	Yes....1<br>No....2<br>Don't know....9	-> Next Q<br>-> Q32<br>-> Q32
28. **How long after birth was (NAME) first put to the breast?**<br>*If respondent reports she put the infant to the breast immediately after birth, circle '00' for 'immediately'.*<br>*If less than 1 hour, circle 'A' for hours and record '00' hours*<br>.<br>*If less than 24 hours, circle 'A' and record number of completed hours, from 01 to 23.*<br>*If 24 hours or longer, circle 'B' and record number of completed days*<br>*If respondent does not know, circle "99".*	Immediately .... 00<br>or<br>A. Hours .... ☐☐<br>or<br>B. Days.... ☐☐<br>Don't know 99	
29. **Is (NAME) still being breastfed?**	Yes....1<br>No....2<br>Don't know.... 9	-> Next Q<br>-> Q32<br>-> Q32
30. **Was (NAME) breastfed yesterday during the day or at night?**	Yes....1<br>No....2<br>Don't know....9	
31. **Sometimes babies are fed breast milk in different ways, for example by spoon, cup or bottle. This can happen when the mother cannot always be with her baby. Sometimes babies are breastfed by another woman, or given breast milk from another woman by spoon, cup or bottle or some other way.**<br>**This can happen if a mother cannot breastfeed her own baby.**<br>**Did (NAME) consume breast milk in any of these ways yesterday during the day or at night?**	Yes....1<br>No....2<br>Don't know....9	

32. **Next I would like to ask you about some liquids that (NAME) may have had yesterday during the day or at night.**<br><br>**Did (NAME) have any (**item from the list**)?:**<br>*Read the list of liquids starting with 'Plain Water'*<br>*Ask the mother to mention and tick from the list*	Yes	No	DK	**33. How many times yesterday during the day or at night did (NAME) eat or drink any (**item from list**)?**<br><br>*Fill in B & C if "Yes" to Q32*
**A. Plain water?**	1	2	9	
**B. Infant formula such as Lactogen, SMA gold/progressive/white, Guigoz 1 or 2, Nan, or Baby milk in tins or sachets?**	1	2	9	B. .... ☐☐
**C. Milk (such as tinned, powdered, or fresh animal milk) or Yogurt?**	1	2	9	C. .... ☐☐
**D. Juice or juice drinks?**	1	2	9	
**E. Clear broth (rice water, banana water)?**	1	2	9	
**F. Thin porridge (e.g. Light Pap)?**	1	2	9	
**G. Liquids such as sweet tea, herbal tea, or soda (e.g. coca cola, fanta), drinks, palm wine?**	1	2	9	
**H. Vitamin or mineral supplements or any medicines?**	1	2	9	
**I. ORS (oral rehydration solution)?**	1	2	9	
**J. Any other liquids?**	1	2	9	

34. **Please describe everything that (NAME) ate yesterday during the day or night, whether at home or outside the home.**

a) Think about when (NAME) first woke up yesterday. Did (NAME) eat anything at that time? if yes: Please tell me everything (NAME) ate at that time.
Probe:**"Anything else?"***until respondent says nothing else. If no, continue to question b).*

b) What did (NAME) do after that? Did (NAME) eat anything at that time? If yes: Please tell me everything (NAME) ate at that time.
*Probe:***"Anything else?"***until respondent says nothing else*.
Repeat question b) above until respondent says the child went to sleep until the next day.
If respondent mentions mixed dishes like a porridge, sauce or stew, probe:

c) **What ingredients were in that (mixed dish)?**
*Probe:***"Anything else?"***until respondent says nothing else.*

As the respondent recalls foods, underline the corresponding food on the next page and circle '1' in the column next to the food group. If the food is not listed in any of the food groups below, write the food in the box labeled 'other foods'. If foods are used in small amounts for seasoning or as a condiment, include them under the condiments food group. Once the respondent finishes recalling foods eaten, read each food group where '1' was not circled, ask the following question and circle '1' if respondent says yes, '2' if no and '9' if don't know:

Yesterday during the day or night, did (NAME) drink/eat any (food group items not already marked 1)?

Other foods:

	Yes	No	DK
**A. Corn/maize, rice, sorghum, millet, or other foods made from these grains or any other grains (e.g. bread, noodles, porridge or other grain products, other local grains)**	1	2	9
**B. Pumpkin, carrots, squash, or orange peteteh?**	1	2	9
**C. Irish potatoes, white sweet potatoes, cassava, yam, garri, or any other foods made from roots?**	1	2	9
**D. Any dark green leafy vegetables (e.g. potato leaves, cassava leaves, krian-krain, green, or moringa)?**	1	2	9
**E. Ripe mango, ripe pawpaw, guava, or water melon?**	1	2	9
**F. Lemon, lime, grapefruit, orange, pineapple, banana, or plantain?**	1	2	9
**G. Any other fruits or vegetables (e.g. Pear, okroh, giblox, jakato, ball tamatis, or seed tamatis)?**	1	2	9
**H. Liver, kidney, heart, or other organ meats?**<br><br>*If "Yes", confirm with respondent that child actually consumed meats and not just sauce cooked with meat. If only sauce consumed, mark "No".*	1	2	9
**I. Any meat, such as beef, pork, lamb, goat, chicken, or duck?**<br><br>*If "Yes", confirm with respondent that child actually consumed meats and not just sauce cooked with meat. If only sauce consumed, mark "No".*	1	2	9
**J. Eggs**	1	2	9
**K. Fresh or dried fish, oysters, crabs, shrimp, cray fish or other seafood?**<br><br>*If "Yes", confirm with respondent that child actually consumed fish etc, and not just sauce cooked with fish etc. If only sauce consumed, mark "No".*	1	2	9
**L. Any foods made from black eye binch, konsho binch, broad binch beans, grandnat, kushu, cowpea, benni, egusi, soya bean, lentils or any other seeds?**	1	2	9
**M. Cheese, yogurt, or other milk products?**	1	2	9
**N. Any oil, fats, or butter, or foods made with any of these?**	1	2	9
**O. Any sugary foods such as chocolates, sweets, candies, pastries, cakes, or biscuits?**	1	2	9
**P. Condiments for flavor, such as pepper, hot pepper, onions, spices, herbs, or fish powder**	1	2	9
**Q. Snails or insects**	1	2	9
**R. Foods made with red palm oil, red palm nut, or red palm nut pulp sauce**	1	2	9
	If all 'NO', go to Q35<br>If at least 1 'YES' or all 'DK' go to Q36		

35. **Did (NAME) eat solid or semi-solid (soft, mushy) food yesterday (e.g. soft cooked rice, cooked potatoes, plasaspenmahun), during the day or night?**<br><br>*If YES for this question and all foods in question 34 above = NO, go back to probe.*	Yes..1<br><br>No..2<br>Don't know 9	->See instructions under Q35<br><br>->Q37<br>->Q37
36. **How many times did (NAME) eat solid or semi-solid (soft, mushy) food yesterday (e.g. soft cooked rice, cooked potatoes, plasaspenmahun), during the day or night?**	Number of times......... ☐☐	
37. **Yesterday, during the day or night, did (NAME) drink anything from a bottle with a nipple?**	Yes..1<br>No..2<br>Don't know 9	

NOTE: *Include the following questions for* ALL *children 0 – 59 months of age:*

Now I would like to ask you about some additional foods and medicines (NAME) may have recently received.

38. **Now I would like to ask you about some particular foods (NAME) may have eaten. I am interested in whether your child had the item even if it was combined with other foods.**<br><br>**Yesterday, during the day or night, did (NAME) consume any iron-fortified cookies or other foods which have added iron (e.g. Bennimix, Cerelac, Golden country, Nutrilac, Frescocem?**<br><br>*If "Yes", ask mother or care giver to show the package of the food for the interviewer to confirm*	Yes..1<br>No..2<br>Don't know 9	
39. **Yesterday, during the day or night, did (NAME) consume any Ready-to-use Therapeutic Food (RUTF) (e.g. Granat)?**	Yes..1<br>No..2<br>Don't know 9	
40. **Yesterday, during the day or night, did (NAME) consume any infant formula containing extra iron, such as Guigoz, Lactogen, SMA, Nan etc?**	Yes..1<br>No..2<br>Don't know 9	
41. **During the last six months was (NAME) given any iron tablets or syrup?**<br><br>*Show iron tablets and syrup*.	Yes..1<br>No..2<br>Not sure if it was iron .... 3<br>Don't know 9	-> Next Q<br>->Q45<br>->Q45<br>->Q45
42. **For how long did (NAME) take iron tablets or syrup?**	One week or less ....1<br>More than 1 week<br>less than 1 month...................2<br>1 month or more.....................3<br>Don't know 9	
43. **Is (NAME) still taking iron tablets or syrup?**	Yes..1<br>No..2<br>Don't know 9	-> Q45<br>-> Next Q<br>->Q45
44. **When did (NAME) stop taking iron tablets or syrup?**	Less than 3 months ago.........1<br>3 months ago or more............2<br>Don't know 9	
45. **During the last six months was (NAME) given a vitamin A capsule?**<br><br>*Show vitamin A capsule.*	Yes..1<br>No..2<br>Not sure if it was vitamin A.....3<br>Don't know 9	
46. **During the last six months was (NAME) given any multi-vitamins, such as Abedic or Alion?**<br><br>*Show capsule or example packages.*	Yes .... 1<br>No .... 2<br>Not sure if it was multi-vitamin...................................3<br>Don't know 9	
47. **During the last six month, was (NAME) given any drug for intestinal worms, such as vermox/Zentel/Albendazole/mebendazole during the last *mami n pekinwel-bodi* week?**	Yes .... 1<br>No .... 2<br><br>Don't know 9	

Comments about data collection with this child:

The form was reviewed by: ______________________________ Date: ______________
Team leader's signature

Data entry clerk name: ______________________ Data entry clerk code number: ☐

48. Written Consent for blood collection:

Now that we've finished the questions, we would like to draw a small amount of (child's name)'s blood. This small blood sample will be used to test for anemia and malaria. In addition, a small portion of blood will be collected to test for other vitamin problems, such as iron and vitamin A.

The anemia and malaria results will be provided in less than 15 minutes following the taking of blood. Should (child's name) be diagnosed with anemia or malaria, we will provide you with a referral slip to get adequate treatment at the nearest health facility. Other results (e.g. iron, vitamin A) will be used by national health officials to better understand the nutrition situation of children in Sierra Leone. All the information will be kept confidential and personal identities will not be revealed in any report.

Blood will be collected by trained technicians and they will use clean and sterile material. The risk for (child's name) is very minimal. At most there will be temporary discomfort where the blood will be taken. The blood draw will take less than 5 minutes.

The participation of (child's name) is entirely voluntary. You may choose not to participate and you may withdraw (child's name)'s participation or that of any household member at any time. Refusal to participate will not involve a penalty or loss of benefits of any kind.

Contact information: If you have any questions or concerns about this study or if any problems arise, please contact the field workers. You may also wish to contact the MoHS Nutrition Programme Director, Aminata Shamit Koroma, directly at 033 705866.

**Do you agree to have *(child's name)* participate in this part of the survey?**<br><br>**If "Yes", can you please sign this page?**	Yes....................1<br><br>No.....................2	------------------------------<br>Mother's or caretaker's name (print)	------------------------------<br>Mother's or caretaker's signature or fingerprint

****Note:** If respondent agrees to participate in the blood collection component of the survey, complete the heading of the following page and write below the location of the laboratory site. If the respondent does not agree to participate, end the interview.

Please take this form to the laboratory site.
This is located at:

__Write location of site here__

Affix HOUSEHOLD label here (starts with "H")	Cluster number ☐☐	Household number ☐☐	Child number ☐☐	Affix CHILD label here (starts with "C")

Address: ___________________ Family name: ___________________ Child's name: ____________________

What is the age of the child?	0 – 5 months 1	-> DO NOT collect blood
	6 - 12 months 2	-> Collect blood from HEEL
	13 – 59 months 3	-> Collect blood from FINGER

Now we would like to take some blood from (NAME)'s heel (6-12 months) or finger (13-59 months)?

49. Hemoglobin concentration	Hb (g/dL)................................☐☐.☐	
50. Approximate volume of blood collected (µL)	µL...☐☐.☐<br>No blood, unsuccessful blood draw . use 88.8	
51. Malaria status from rapid test kit	Positive .... 1<br>Negative 2	
52. Phlebotomist's code number:	Code number...................................☐☐	

Comments about blood collection of this child:

The form was reviewed by: ____ Date: ________________
Team leader's signature

Data entry clerk name: _______________________________ Data entry clerk code number: ☐

Affix HOUSEHOLD label here (starts with "H")	**WOMAN QUESTIONNAIRE**<br>**SIERRA LEONE NATIONAL MICRONTURIENT SURVEY 2013**	Affix WOMAN label here (starts with "W")

1. Cluster number .... ☐☐	2. HH number<br>(in cluster control form) .... ☐☐
3. Name of this woman:________________________	4. Woman's line number..... ☐☐
5. Was this woman randomly selected? Yes................. 1<br>No................. . 2<br>*Check Box A or C on page 3 of household questionnaire*	6. Interviewer number......... ☐☐

7. Date of data collection .... ☐☐ / ☐☐ / ☐☐ (Day / Month / Year)

8. Final result of woman data collection .... (enter code from below) ☐

FINAL RESULT CODES:

Completed interview and accepted participation in either urine and/or blood collection 1	Refused interview and all data collection 3
	Woman not home or not available...................... 4
Completed interview and refused participation in both urine/blood collection 2	Other (specify) ________________________ .. 8

9. Number of urine specimens requested from woman?	
At end of the interview, provide two urine beakers to the first four women <u>in each cluster</u> to complete a woman's questionnaire. The first beaker (with yellow label) should be filled immediately, and the second beaker (with red label) approximately 24 hours later.	Number of specimens requested............. 1
All other women in the cluster should receive <u>only</u> one urine beaker.	Number of specimens requested............. 2

Repeat greeting if not already read to this respondent:<br><br>**We are working with the Ministry of Health and Sanitation in Sierra Leone. We are conducting a national nutrition survey to better understand various types of nutritional problems such as anemia, vitamins and minerals in women and children. This information will help the government to plan for better health in the future.**	*If greeting at the beginning of the household questionnaire has already been read to this woman, then read the following:*<br><br>**Now I would like to talk to you more about YOUR health and other topics. All the information we obtain will remain strictly confidential and your answers will never be shared with anyone other than our project team.**<br><br>**The survey usually takes about 20 minutes to complete, and includes answering questions and a visit to (location of lab/measuring site) to to take a small blood sample.**

10. May I start now?

- ☐ YES, PERMISSION IS GIVEN -> BEGIN THE INTERVIEW.
- ☐ NO, PERMISSION IS NOT GIVEN -> COMPLETE THIS COVER PAGE. DISCUSS THIS RESULT WITH YOUR TEAM LEADER.

I would first like to ask you some questions about yourself.

11. **In what month and year were you born?**	Month .... ☐☐<br>Year .... ☐☐☐☐	Don't know, enter '99' or '9999'
12. **How old are you?**<br>*Probe:* **How old were you at your last birthday?**<br>*Compare month and year of birth and stated age; correct one if necessary*	Age .... ☐☐<br>(in completed years)<br>(enter '99' if unknown)	
13. **Have you ever attended school?**	Yes ....1<br>No...2<br>Don't know 9	->NEXT Q<br>-> Q15<br>-> Q15
14. **What is the highest level of school you <u>attended</u>?**<br><br>**How many years at this level did you <u>complete</u>?**	See table below	
15. **What does this say?**<br><br>*Show sentence on the card to the respondent. If respondent cannot read whole sentence, probe:*<br><br>**Can you read part of the sentence to me?**	Cannot read at all......................................1<br>Able only to read only parts of sentence....2<br>Able to read whole sentence......................3<br>No sentence in required language<br>(specify)__________________ ....4<br>Blind, mute, visually/speech impaired........5	
16. **What is your marital status now?**	Never married, never lived<br>with a man ....1<br>Currently married ....2<br>Living with a man, but not<br>married ....3<br>Divorced ....4<br>Separated ....5<br>Widowed 6	
17. **What is your job outside the home?**	No job .... 0<br>Unskilled labor .... 1<br>Skilled labor .... 2<br>Agriculture .... 3<br>Shop or office .... 4<br>Own business .... 5<br>Professional .... 6<br>Other (specify: ______________) ....8<br>Don't know 9	
18. **Do you smoke cigarettes?**	Yes ....1<br>No...2	-> Next Q<br>-> Q20
19. **On average, how many cigarettes do you smoke per day?**	Number .... ☐☐	

Question 14:

	Circle code	# years completed	DK
Kindergarten	0	0 1 2 3	9
Primary	1	0 1 2 3 4 5 6 7	9
JSS-Junior Secondary ...	2	0 1 2 3	9
SSS-Senior Secondary...	3	0 1 2 3	9
Vocational/ commercial/ nursing/ technical/ teaching	4	0 1 2 3	9
Tertiary/college/univ........	5	0 1 2 3 4 5 6	9
Don't know	9		

20. **Are you pregnant now?**	Yes ....1<br>No .... 2<br>Unsure/ don't know 9	-> Next Q<br>-> Q22<br>-> Q22
21. **How many months pregnant are you?**	Number of months	
22. **How many times, in total, have you been pregnant?**<br>*If pregnant now, include this pregnancy.*<br>*If never pregnant, enter "00".*	Number of times ....<br>Don't know 99	00->Q26
23. **During your last pregnancy, did you take iron or folic acid supplements for 90 days or more?**	Yes ....1<br>No .... 2<br>Unsure/ don't know 9	
24. **How many times, in total, have you given birth to a baby?** *Include still births and live births*	Number of times	00->Q26
25. **Are you currently breastfeeding a child?**	Yes...1<br>No...2	

26. **Please describe everything that you ate yesterday during the day or night, whether at home or outside the home.**

a) Think about when you first woke up yesterday. Did you eat anything at that time? if yes: Please tell me everything that you ate at that time.
Probe: **"Anything else?"** *until respondent says nothing else. If no, continue to question b).*

b) What did you do after that? Did you eat anything at that time? If yes: Please tell me everything you ate at that time.
Probe: **"Anything else?"** *until respondent says nothing else.*
Repeat question b) above until respondent says she went to sleep until the next day.
If respondent mentions mixed dishes like a porridge, sauce or stew, probe:

c) What ingredients were in that (mixed dish)?
Probe: **"Anything else?"** *until respondent says nothing else.*

As the respondent recalls foods, underline the corresponding food and circle '1' in the column next to the food group. If the food is not listed in any of the food groups below, write the food in the box labelled 'other foods'. If foods are used in small amounts for seasoning or as a condiment, include them under the condiments food group. Once the respondent finishes recalling foods eaten, read each food group where '1' was not circled, ask the following question and circle '1' if respondent says yes, '2' if no and '9' if don't know:

Yesterday during the day or night, did you drink/eat any (food group items not already marked 1)?			
Other foods:			
	Yes	No	DK
**A. Corn/maize, rice, sorghum, millet, or other foods made from these grains or any other grains (e.g. bread, noodles, porridge or other grain products, other local grains)**	1	2	9
**B. Pumpkin, carrots, squash, or orange peteteh?**	1	2	9
**C. Irish potatoes, white sweet potatoes, cassava, yam, garri, or any other foods made from roots?**	1	2	9
**D. Any dark green leafy vegetables (e.g. potato leaves, cassava leaves, krian-krain, green, or moringa)?**	1	2	9
**E. Ripe mango, ripe pawpaw, guava, or water melon?**	1	2	9
**F. Lemon, lime, grapefruit, orange, pineapple, banana, or plantain?**	1	2	9
**G. Any other fruits or vegetables (e.g. Pear, okroh, giblox, jakato, ball tamatis, or seed tamatis)?**	1	2	9
**H. Liver, kidney, heart, or other organ meats?**<br><br>If "Yes", confirm that respondent actually consumed meats and not just sauce cooked with meat. If only sauce consumed, mark "No".	1	2	9
**I. Any meat, such as beef, pork, lamb, goat, chicken, cat, dog, monkey or duck?**<br><br>If "Yes", confirm that respondent actually consumed meats and not just sauce cooked with meat. If only sauce consumed, mark "No".	1	2	9
**J. Eggs**	1	2	9
**K. Fresh or dried fish, oysters, crabs, shrimp, or cray fish or other seafood?**<br><br>If "Yes", confirm that respondent actually consumed fish etc, and not just sauce cooked with fish etc. If only sauce consumed, mark "No".	1	2	9
**L. Any foods made from black eye binch, konsho binch, broad binch beans, grandnat, kushu, cowpea, benni, egusi, soya bean, lentils or any other seeds?**	1	2	9
**M. Cheese, yogurt, or other milk products?**	1	2	9
**N. Any oil, fats, or butter, or foods made with any of these?**	1	2	9
**O. Any sugary foods such as chocolates, sweets, candies, pastries, cakes, or biscuits?**	1	2	9
**P. Condiments for flavor, such as pepper, hot pepper, onions, spices, herbs, or fish powder**	1	2	9
**Q. Snails or insects, snakes**	1	2	9
**R. Foods made with red palm oil, red palm nut, or red palm nut pulp sauce**	1	2	9

Now I would like to ask you about some foods which may contain extra nutrients.

27. **Have you heard about iodized salt (i.e. salt with added iodine)?**	Yes .... 1<br>No .... 2<br>Don't know 9	-> Next Q<br>-> Q29<br>-> Q29
28. **Why do you think iodized salt is important?**<br><br>*Do not prompt. Mark all responses mentioned*	Prevents goitre/Gehgeh....... A<br>Improves intelligence .... B<br>Prevents iodine deficiency .. C<br>Improve health status .... D<br>Other .... Y<br>(specify: _____________)<br>Don't know .... Z	
29. **Have you heard about fortified vegetable oil (i.e. oil with nutrients added)?**	Yes .... 1<br>No .... 2<br>Don't know 9	-> Next Q<br>-> Q31<br>-> Q31
30. **Why do you think fortified oil (i.e. with added nutrients) is important?**<br><br>*Do not prompt. Mark all responses mentioned*	Prevents blindness .... A<br>Reduces mortality .... B<br>Prevents vitamin deficiency C<br>Improve health status .... D<br>Other .... Y<br>(specify: _____________)<br>Don't know .... Z	

Now I would like to ask you some questions about vitamins or minerals you may be taking or have recently taken.

31. **During the last six months did you take any iron tablets or syrup, such as Dexorange, Dawn of Life, Pinoplex, Haemoforte, Rescofer Blood Tonic, HB 12?** *Show iron tablets and syrup.*	Yes .... 1<br>No .... 2<br>Not sure if it was iron 9	-> Next Q<br>-> Q35<br>-> Q35
32. **For how long did you take iron tablets or syrup?**	One week or less .... 1<br>More than 1 week, less than 1 month .... 2<br>One month or more 3	
33. **Are you still taking iron tablets or syrup?**	Yes .... 1<br>No 2	-> Q35<br>-> Next Q
34. **When did you stop taking iron tablets or syrup?**	Less than 3 months ago .... 1<br>3 months ago or more .... 2<br>Don't know 9	
35. **During the last six months did you take any folic acid tablets?** *Show folic acid tablets*.	Yes .... 1<br>No .... 2<br>Not sure if it was folic acid 9	-> Next Q<br>-> Q39<br>-> Q39
36. **For how long did you take folic acid tablets?**	One week or less .... 1<br>More than 1 week, less than 1 month .... 2<br>One month or more 3	
37. **Are you still taking folic acid tablets?**	Yes .... 1<br>No 2	-> Q39<br>-> Next Q
38. **When did you stop taking folic acid tablets?**	Less than 3 months ago .... 1<br>3 months ago or more .... 2<br>Don't know 9	
39. **Following your last pregnancy (i.e. after delivery), did you take any vitamin A capsules?** *Show vitamin A capsule.*	Yes .... 1<br>No .... 2<br>This woman has never been pregnant .... 3<br>Not sure if it was vitamin A 9	
40. **During the last six months did you take any multi-vitamin supplements, such as Fefol, Vitamin B complex, Omega H3?**	Yes .... 1<br>No .... 2<br>Not sure if it was a multi-vitamin 9	->CONSENT<br>-> Consent<br>-> Consent

Comments about data collection with this woman:

The form was reviewed by: ______________________ Date: ____________

Team leader's signature

Data entry clerk name: ______________________ Data entry clerk code number: ☐

41. Written Consent for urine and/or blood collection:

Now that we've finished the questions, we would like to draw a small amount of blood. This small blood sample will be used to test for anemia and malaria. In addition, a small portion of blood will be collected to test for other vitamin problems, such as iron, vitamin A, folate, vitamin B12 and iodine. Also, we would like to ask you to provide a small amount of urine, which will also be used for iodine testing.

The anemia and malaria results will be provided in less than 15 minutes following the taking of blood. Should you be diagnosed with severe anemia or malaria, we will provide you with a referral slip to get adequate treatment at the nearest health facility. Other results (e.g. iron, vitamin A, etc) will be used by national health officials to better understand the nutrition situation of women in Sierra Leone. All the information will be kept confidential and personal identities will not be revealed in any report.

Blood will be collected by trained technicians and they will use clean and sterile material. The risk for you is very minimal. At most there will be temporary discomfort where the blood will be taken. The blood draw will take less than 5 minutes.

Your participation is entirely voluntary. You may choose not to participate and you may withdraw your participation or that of any household member at any time. Refusal to participate will not involve a penalty or loss of benefits of any kind.

Contact information: If you have any questions or concerns about this study or if any problems arise, please contact the field workers. You may also wish to contact the MoHS Nutrition Programme Director, Aminata Shamit Koroma, directly at 033 705866.

**Do you agree to participate in this part of the survey?**<br><br>If "Yes", can you please sign this page?**	Yes....................1<br><br>No 2	______________________<br>Woman's name (print)	______________________<br>Woman's signature or fingerprint

****Note:** If respondent agrees to provide urine only, blood only, or both urine and blood, circle "Yes" for Q41 above and complete the heading of the following page. Then give the respondent the labelled urine beaker and write below the location of the laboratory site. If the respondent does not agree to participate, end the interview.

Please take this form and the filled urine beaker to laboratory site. This is located at:

__Write location of site here__

Affix HOUSEHOLD label here (starts with "H")	Cluster number ☐☐	Household number ☐☐	Woman number ☐☐	Affix WOMAN label here (starts with "W")

Address: ____________________ Family name: __________________ Woman's name: ________________

Is this woman pregnant?	Yes..1<br><br>No 2	->Take blood from finger and mark 9.9 for Q45<br><br>->Take blood from vein

Pregnant women: **Now we would like to do a fingerpick to measure anemia and malaria.**

Non-pregnant women: **Now we would like to take some blood from your vein for testing for vitamin levels.**

42. Urine beaker received?	Yes ....1<br>No 2	
43. Hemoglobin concentration	Hb (g/dL) .... ☐☐.☐	
44. Malaria status from rapid test kit	Positive ....1<br>Negative 2	
45. Approximate volume of blood collected (ml)	ml .... ☐.☐<br><br>No blood, unsuccessful blood draw ..... use 8.8<br>No blood, pregnant woman................. use 9.9	
46. Phlebotomist's code number:	Code number .... ☐☐	

Comments about data collection with this woman:

The form was reviewed by: ____ Date: ________________

Team leader's signature

Data entry clerk name: _________________________ Data entry clerk code number: ☐
